# Local variation in musculoskeletal pain consultation rates in primary care: findings from an ecologic study in Staffordshire

**DOI:** 10.1017/S1463423625100133

**Published:** 2025-06-25

**Authors:** George M. Peat, Jonathan C. Hill, Dahai Yu, Simon Wathall, Emma Parry, James Bailey, Kay Stevenson, Clare Thompson, Ross Wilkie, Krysia Dziedzic, Kelvin P. Jordan, the MIDAS Patient Advisory Group

**Affiliations:** 1 Professor of Clinical Epidemiology, Centre for Applied Health & Social Care Research, Sheffield Hallam University, Sheffield, UK; 2 Visiting Professor, Primary Care Centre Versus Arthritis, School of Medicine, Keele University, Staffordshire, UK; 3 Professor of Physiotherapy, Primary Care Centre Versus Arthritis, School of Medicine, Keele University, Staffordshire, UK; 4 Senior Research Fellow, Primary care Centre Versus Arthritis, School of Medicine, Keele University, Staffordshire, UK; 5 Health Informatics Specialist, Keele Clinical Trials Unit, School of Medicine, Keele University, Staffordshire, UK; 6 NIHR Academic Clinical Lecturer in Primary Care, Primary Care Centre Versus Arthritis, School of Medicine, Keele University, Staffordshire, UK; 7 Data Manager, School of Medicine, Keele University, Staffordshire, UK; 8 Consultant Physiotherapist, Midlands Partnership University NHS Foundation Trust, Staffordshire, UK; 9 Honorary Professor, Clinical Care and Leadership, School of Medicine, Keele University, Staffordshire, UK; 10 Trial Manager, Keele Clinical Trials Unit, School of Medicine, Keele University, Staffordshire, UK; 11 Professor Public Health and Epidemiology, Primary Care Centre Versus Arthritis, School of Medicine, Keele University, Staffordshire, UK; 12 Professor of Musculoskeletal Therapies, Primary Care Centre Versus Arthritis, School of Medicine, Keele University, Staffordshire, UK; 13 Impact Accelerator Unit, Keele University, Staffordshire, UK; 14 Professor of Biostatistics Primary Care Centre Versus Arthritis, School of Medicine, Keele University, Staffordshire, UK.

**Keywords:** electronic health record, musculoskeletal, primary care

## Abstract

Variation between general practices in the rate of consultations for musculoskeletal pain conditions may signal important differences in access to primary care, perceived usefulness, or available alternative sources of care; however, it might also just reflect differences in underlying ‘need’ between practices’ registered populations. In a study of 30 general practices in Staffordshire, we calculated the proportion of adults consulting for a musculoskeletal pain condition, then examined this in relation to selected practice and population characteristics, including the estimated prevalence of self-reported musculoskeletal problems and chronic pain in each practices’ registered population. Between September 2021 and July 2022, 18,388 adults were consulted for a musculoskeletal pain condition. After controlling for length of recruitment, time of year, and age-sex structure, the proportion consulting varied up to two-fold between practices but was not strongly associated with the prevalence of self-reported long-term musculoskeletal problems, chronic pain, and high-impact chronic pain.

## Background

The move towards place-based health and care systems in England has accelerated the need for local decision-makers to have accurate, trustworthy, and meaningful information at increasingly granular levels to target policies and actions (Department of Health & Social Care 2022; UK Government 2022). The prevention and management of common, disabling musculoskeletal pain conditions like back pain, neck pain, and osteoarthritis represents a growing challenge for health systems (McKee *et al.*, 2021) particularly in community and primary care settings where most assessment and (self-)management takes place (Hobbs *et al.*, 2016) and where relevant data have historically been absent, limited, or fragmented.

Significant variation between practices in the rate of primary healthcare contacts may indicate problems with access. However, variation may also reflect genuine differences in the underlying prevalence of MSK pain conditions in practice populations (Versus Arthritis/Imperial College London, 2023; Lynch *et al.*, 2023). To make sense of this, information from multiple sources needs to be brought together to ‘complete the clinical picture’ (Morris 1955, Hannay 1979, Hart 1992). A study by Walsh et al.,(Walsh *et al.*, 1992) conducted over 20 years ago, appears unique in having achieved this specifically for musculoskeletal pain conditions in UK primary care. In their cross-sectional survey of 3667 adults aged 20–59 years randomly sampled from the practice lists of 136 general practitioners located in 8 areas around Britain, they found remarkably little difference between areas in the prevalence of low back pain. In contrast, the threshold for consulting general practice varied three- to four-fold even after adjusting for age, sex, occupational class, and severity of symptoms. Differences in recording practices did not appear to explain the findings and the authors speculated on the role of variation in the perceived benefits of consultation or in the accessibility of alternative sources of care.

We wanted to see if an approach within an integrated care system could be developed using currently available data sources and, if so, to what extent the pattern of findings from Walsh et al. might still hold. To address this, and as part of a wider programme of research on the enrichment and integration of data for local musculoskeletal population health intelligence, we designed and conducted a study within a single integrated care system.

## Methods

This was a descriptive, cross-sectional, ecologic study at the level of general practices (GP).

### Setting

The MIDAS-GP study was set in the area of North Staffordshire & Stoke-on-Trent within Staffordshire Integrated Care System. The area covers three local authorities and at the time of the study was served by 70 general practices within 13 Primary Care Networks.

### Participating general practices

General practices were eligible to participate if they used a practice computer system and text-messaging service suitable for patient identification and recruitment, and were willing and able to undertake anonymised medical record audits of musculoskeletal (MSK) consultations. We sought to involve at least 26 general practices covering all of the 13 Primary Care Networks in the area and attempted to over-sample practices located in more socioeconomically deprived communities and those serving the most ethnically diverse populations.

### Data collection

Staff from NIHR Clinical Research Network: West Midlands worked with practice staff to perform weekly/fortnightly searches of the primary care electronic health record to identify all consecutive adults aged 18 years and over presenting to the practice during the recruitment period for a MSK pain condition based on a pre-defined list of 498 SNOMED CT concept IDs (Jordan *et al.*, 2021), Supplementary Data A). Between September 2021 and July 2022, recruitment was staggered across practices to manage finite resources to support recruitment. Practices were advised that the recruitment period would last for three to six months.

### Practice-level covariates

Selected potential practice-level determinants of the rate of musculoskeletal consultations were identified and extracted from a range of publicly available sources. In addition, we sent a brief online questionnaire to practices to collect information on practice characteristics and specific MSK services not available from routine publicly available sources (Supplementary Data B).

#### Estimates of population ‘need/burden’

Population prevalence estimates of MSK pain conditions in the registered practice populations were obtained from three sources: (i) The GP Patient Survey 2022 provided weighted prevalence estimates of self-reported arthritis or a long-term back or joint problem in adults aged 16 years and over (source: GP Patient Survey (gp-patient.co.uk)). (ii) A local general population survey conducted in 2017 provided neighbourhood-level modelled prevalence estimates of self-reported chronic pain and high-impact chronic pain in adults aged 35+ years (Lynch *et al.*, 2023) From these, we derived practice-specific prevalence estimates (see Supplementary Data C). (iii) The Quality and Outcomes Framework 2021–2022 provided estimates of the prevalence of obesity for all registered adults aged 18+ years in each practice.

#### Registered population characteristics

We extracted the total size of the registered population and its age and sex distribution at the mid-period of recruitment for each practice. Modelled estimates of the proportion of the registered practice population from Black, Asian, and minority ethnic backgrounds were also extracted, together with weighted practice-level deprivation scores.

#### Practice organization and performance characteristics

To reflect potential access- and capacity-related determinants, we extracted information on practice size, and indicators of clinical staff time per 10,000 patients, overall practice performance (Quality and Outcomes Framework (QOF) overall achievement score, independent Care Quality Commission inspection overall rating – latest available) and patient experience (percentage reporting positive experience of practice from General Practice Patient Survey (2022). From the short online questionnaire to practices, we obtained a count of the number of selected services available to patients with a MSK pain condition (0–11), and the number of different types of musculoskeletal clinical decision support systems each practice reported using (0–8).

### Statistical analysis

For each practice, we calculated the crude proportion of registered adults aged 18 years and over consulting and receiving a relevant SNOMED CT code at least once during the practice recruitment period. The denominator was the registered population aged 18 years and over at the midpoint of each practice’s recruitment period.

The proportion of adults consulting for a MSK pain condition will increase as the recruitment period increases and seasonal variation in consultation rates for MSK conditions was expected. Therefore, we plotted the proportion of adults consulting for a MSK pain condition against the length of recruitment period controlling for whether the practice was open during December. We then explored potential determinants of variation with scatterplots and using fractional polynomials to select the best-fitting relationship with each covariate after controlling for: length of recruitment period, whether the practice was recruiting in December, the proportion of the registered population who were female, and the proportion of the registered population aged 65 years and over. Likelihood ratio tests were used to examine whether each practice-level determinant explained variation in the proportion consulting compared to a model with just the above four covariates.

Three sensitivity analyses: (1) repeated the above analyses but excluded MSK pain consulters with an inflammatory disease code recorded in the previous three years; (2) replaced the single point estimate from the General Practice Patient Survey in 2022 with the average of estimates from 2021, 2022, and 2023; (3) additionally adjusted for the practice-specific rate of appointments per 1000 patients (available and extracted from NHS Digital GP Appointments Data and used a proxy for the completeness of recording consultations in practices).

### Patient and public involvement

The MIDAS programme has a dedicated Public Advisory Group (PAG) comprising seven people with lived experience of musculoskeletal conditions drawn from Keele University’s Research User Group. The PAG met with the MIDAS Programme Lead, Chief Investigators, Trial Manager, and other members of the research team on a monthly basis via MS Teams. PAG members advised on the design of the study and interpretation of the findings and suggested revisions to the draft manuscript.

## Results

A total of 30 general practices participated in MIDAS-GP, with at least one practice from each of the 13 local PCNs. Data from one week were excluded for two general practices due to an error in the initial installation and running of the SNOMED CT search function.

### General practice consultation rates for musculoskeletal pain conditions

During the recruitment period, we observed a total of 18,388 adults consulting for a relevant MSK pain condition, equivalent to 9.0% (95%CI: 8.9, 9.1) of all registered adults over a median recruitment period of 126.5 days across the participating practices. The corresponding figures after excluding those with a prior inflammatory disease code were 13,961 or 6.8% (95%CI: 6.7, 6.9) (Supplementary Data D2). The crude proportion of adults consulting for a MSK pain condition varied up to two-fold between practices (Table 1) which remained after accounting for differences in the length of time that practices were open for recruitment (Supplementary Data D1).

**Table 1.** Proportion of adults consulting for a MSK Pain Condition, by GP practice: North Staffordshire & Stoke-on-Trent

GP Practice	Recruitment period (days)	Total registered population 18+^§^	Consulted with MSK Pain code^†^		
			n	Pr	95%CI
GPP020	184	7,083	1,127	0.159	0.151, 0.168
GPP028	197	3,007	440	0.146	0.134, 0.159
GPP021	175	3,646	514	0.141	0.130, 0.153
GPP011	113	11,105	1,391	0.125	0.119, 0.132
GPP016	167	3,203	401	0.125	0.114, 0.137
GPP017	167	2,508	304	0.121	0.109, 0.135
GPP010	198	6,601	700	0.106	0.099, 0.114
GPP015	185	4,351	453	0.104	0.094, 0.114
GPP022	91	11,086	1,133	0.102	0.097, 0.108
GPP009	133	9,137	915	0.100	0.094, 0.106
GPP024	183	3,843	380	0.099	0.090, 0.109
GPP018	119	6,469	615	0.095	0.088, 0.102
GPP019	117	5,658	530	0.094	0.086, 0.102
GPP014	176	7,335	670	0.091	0.085, 0.098
GPP004	120	7,635	669	0.088	0.081, 0.094
GPP029	184	7,695	674	0.088	0.081, 0.094
GPP005	170	5,495	480	0.087	0.080, 0.095
GPP001	110	8,503	690	0.081	0.076, 0.087
GPP003	84	6,167	485	0.079	0.072, 0.086
GPP002	99	7,214	561	0.078	0.072, 0.084
GPP007	105	8,401	650	0.077	0.072, 0.083
GPP026	184	4,690	361	0.077	0.070, 0.085
GPP006	120	9,623	729	0.076	0.071, 0.081
GPP025	111	8,968	666	0.074	0.069, 0.080
GPP027	177	4,625	333	0.072	0.065, 0.080
GPP030	69	2,960	197	0.067	0.058, 0.076
GPP008	70	8,321	547	0.066	0.061, 0.071
GPP012	113	10,091	631	0.063	0.058, 0.067
GPP013	167	10,544	657	0.062	0.058, 0.067
GPP023	83	8,503	485	0.057	0.052, 0.062
Median	126.5	7,149	588	0.088	
TOTAL	4,171	204,467	18,388	0.090	0.089, 0.091

† Unique individuals consulting with a Keele 500 MSK Pain code during the recruitment period at a participating practice

§ Registered population aged 18 years and over at the practice-specific mid-point of recruitment (data source: NHS Digital ‘Patients Registered at a GP Practice’ Patients registered at a GP practice - NHS Digital)

### Relation to population prevalence estimates

Practice-specific population prevalence estimates for a) a long-term MSK condition in adults aged 16 years and over ranged from 13% to 30%, b) chronic pain in adults aged 35 years and over ranged from 24% to 44%, and c) high-impact chronic pain ranged from 9% to 22%.

**Figure 1.**
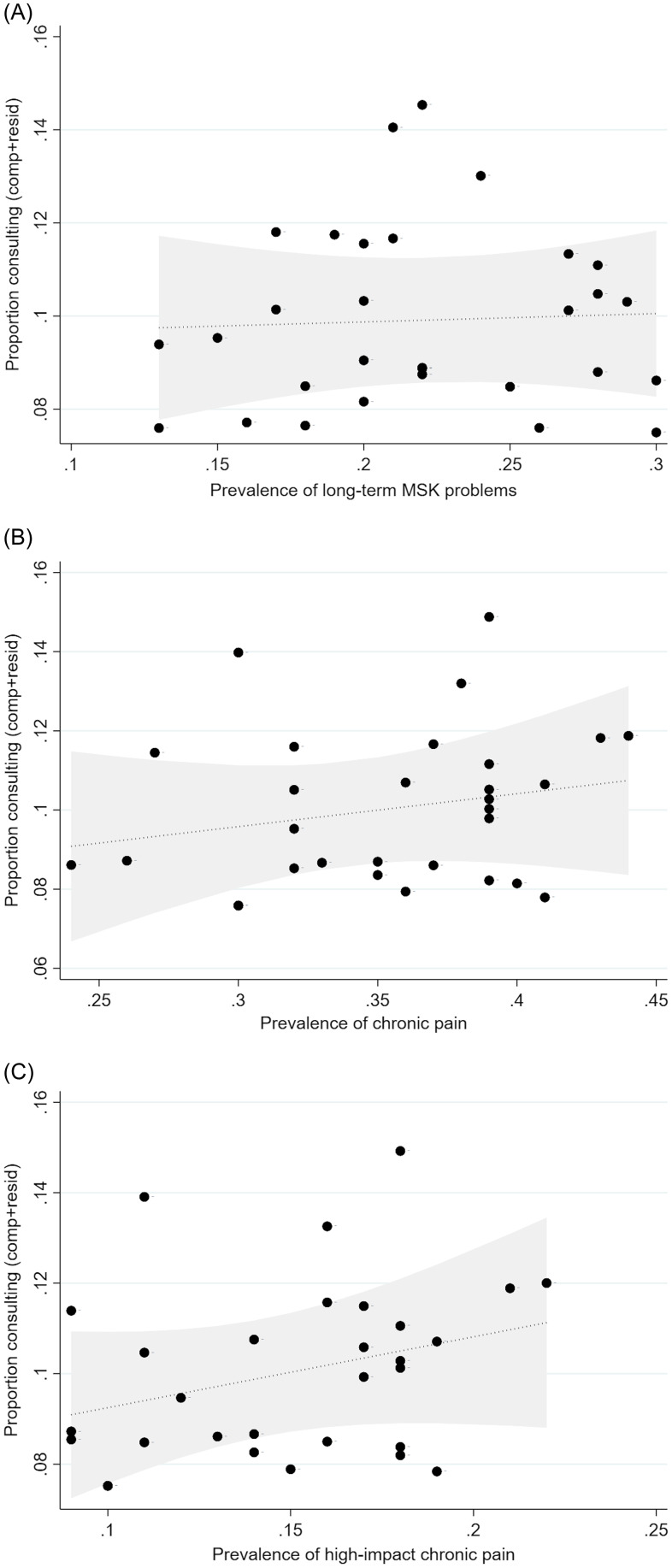
Relationship between consultation for MSK pain conditions and population prevalence estimates. **A.** x-axis = weighted prevalence of arthritis or long-term MSK problem in adults aged 16+ years (GP Patient Survey, 2022). **B.** x-axis = weighted prevalence of chronic pain in adults aged 35+ years (PRELIM, 2017). **C.** x-axis = Weighted prevalence of high-impact chronic pain in adults aged 35+ years (PRELIM, 2017). The y = axis in each figure represents the component + residual value for the proportion consulting for a MSK pain condition. Relationship shown is after adjusting for length of recruitment period, recruitment was open in December, proportion of registered population female, proportion of registered population aged 65+ years.

The crude proportion of adults consulting for a coded MSK pain condition was weakly related to estimates of the population prevalence of MSK conditions or chronic pain among adults registered with the practice (Figure 1).

### Relation to other population and practice characteristics

The relationships between the crude proportion of adults consulting for a coded MSK pain condition and selected population and practice characteristics were also generally weak (Supplementary Data D3). The proportion consulting for a MSK pain condition appeared positively associated with the proportion of the registered practice population that were female. Contrary to expectations, the proportion consulting appeared to be lower in larger practices.

Similarly, weak associations were observed when we excluded consulters for MSK pain who had a previous diagnosis of inflammatory arthritis in their primary care record, when we used the average of three annual estimates of the prevalence of long-term MSK problems, and when we adjusted for appointments per 1000 patients (Supplementary Data D4-D7).

## Discussion

### Summary

This ecologic study of 30 general practices within a single integrated care system in England found that the proportion of adults with a recorded consultation for MSK pain varied up to two-fold between GP practices and that this variation was not strongly related to differences in available estimates of the underlying population prevalence of long-term musculoskeletal conditions or pain.

### Comparison with existing literature

Several UK studies have estimated what proportion of people with low back pain or joint pain/osteoarthritis consult primary care, and investigated factors that may determine this (Adamson *et al.*, 2011; Elliott *et al.*, 2011; Hillman *et al.*, 1996; Jinks *et al.*, 2004; Beyera *et al.*, 2019). Our finding of variation in consultation rates that is not explained by the underlying prevalence of the conditions in the registered population is consistent with Walsh et al (Walsh *et al.*, 1992) although there are several differences in study design that limit direct comparison. The variation in consultation rates in the current study did not appear as great and estimates from the GP Patient Survey and a previous cross-sectional survey suggested quite large variation in the prevalence of self-reported MSK problems or chronic pain in the registered adult population. Random error in practice-specific estimates did not appear to fully account for the findings. However, our study provides relatively few clues on the reasons for between-practice variation in recorded consultation rates for MSK pain conditions. Clear associations were not found between consultation rates and indicators of clinical staffing levels, the level of patient-rated positive experience of the practice in general, QOF score, the range of services available for MSK patients, or the number of relevant MSK decision support systems reportedly used in each practice. Practices with more GPs and nurses have previously been shown to have higher all-cause consultation rates(Mukhtar *et al.*, 2018) and higher all-cause GP consultation rates have been associated with higher rates of patient satisfaction with respect to access(Lay-Flurrie *et al.*, 2019). Taken at face value, our null findings suggest accessibility and quality of MSK services in primary care are not strong determinants of recorded consultation rates specifically for MSK pain in adults. However, our study used relatively crude indicators of these complex underlying constructs: it has been argued, for example, that measures of supply offer a relatively limited view of accessibility, and that other considerations with a wider ‘candidacy’ framework may be needed to understand variability in levels of access (Sinnott *et al.*, 2024). Under-recording of musculoskeletal pain conditions in general practice electronic health records has previously been described(Yu *et al.*, 2018), and variation in the degree of under-recording may contribute to our findings.

Thirty general practices is a small number for an ecologic study and these were based within a single integrated care system. However, our approach is readily scalable and replication across a larger number of practices would be useful, ideally observing consultations over a full calendar year to confidently remove seasonal variation. A larger study involving more general practices and a longer period of observation would be less subject to random error and, depending on the sample frame of general practices used and sampling technique (e.g. random or stratified-random), would produce findings that required fewer assumptions to generalize to all integrated care systems across England. Short-term, acute MSK injuries were included in the consultation count but may not be reflected in prevalence estimates that focus on long-term MSK conditions and pain, thereby producing a spurious ‘mismatch’ between consultation rates and underlying prevalence.

### Implications for research and/or practice

Healthcare service use, including the patient pathway from problem identification to primary care consultation, is determined by a wide variety of contextual factors, individual characteristics, health behaviours and outcomes (Ford *et al.*, 2016; Lederle *et al.*, 2021). A close relationship between consultation and prevalence cannot be expected. Nevertheless, our findings are a reminder that rates of presentation to primary care need not be a reliable indicator of underlying musculoskeletal health in the population. Explanations posited by previous work – differences in accessibility, perceived usefulness, and alternative sources of care and support – remain important to understand equitable and effective care for musculoskeletal pain conditions within local health systems. To inform and influence policies and practices designed to improve accessibility of general practice for MSK pain conditions, we argue for a move beyond cross-sectional studies towards ‘natural experiments’ and other experimental/interventional and mixed-methods study designs that will better support inferences on the effects of changing staffing, resource allocation, and other policies on MSK consultation rates. Candidate interventions include the introduction of first contact physiotherapists in general practice: understanding their effect on MSK consultation rates could be a useful complement to current evaluation projects (Walsh *et al.*, 2024).

## Supporting information

Peat et al. supplementary material Peat et al. supplementary material

## Data Availability

The de-identified, aggregated, practice-level analysis dataset and statistical code are publicly available on Open Science Framework at https://osf.io/e542w/.

## Supplementary material

The supplementary material for this article can be found at https://doi.org/10.1017/S1463423625100133
