# Supplementary material for: Local variation in musculoskeletal pain consultation rates in primary care: findings from an ecologic study in Staffordshire
Source: Prim Health Care Res Dev. 2025 Jun 25;26:e52. doi: 10.1017/S1463423625100133 (PMC12188128; doi:10.1017/S1463423625100133)
Supplement: Peat et al. supplementary material [file S1463423625100133sup001.docx]

**Local variation in musculoskeletal pain consultation rates in primary care: findings from an ecologic study in Staffordshire**

**SUPPLEMENTARY DATA**

Contents

1. SNOMED-CT codelists (pp2-13)
2. List of covariates and sources, including questions from brief online GP practice questionnaire used in the current study (pp14-15)
3. Technical appendix: Method for obtaining practice-specific modelled prevalence estimates of chronic pain and high impact chronic pain (p16)
4. Data tables and scatterplots (pp17-28)

D1. Proportion of adults consulting for a MSK pain condition vs length of recruitment period

D2. Proportion of adults consulting for a MSK Pain Condition, by GP practice: North Staffordshire

& Stoke-on-Trent

D3. Relationships between proportion consulting for MSK pain condition and selected covariates

D4. Sensitivity analysis 1 – exclusion of consulting cases with a previous record of inflammatory

arthritis

D5. Sensitivity analysis 2 – Replacement of single annual prevalence estimate with 3-year average

annual prevalence estimate

D6. Sensitivity analysis 3 – Additional adjustment for recorded number of appointments per 1000

patients as a proxy for completeness of recording consultations

D7. Summary table of associations with covariates

1. **SNOMED-CT Codelists**

## Keele Core Set of SNOMED CT Concept IDs: MSK Pain/Injury (Adult). Version 1.0, 2021-10-14

| **SnomedCTConceptId** | **Preferred term** | **Body region** |
| --- | --- | --- |
| 1003721002 | Pain of joint of knee | KNE |
| 1003722009 | Pain of knee region | KNE |
| 10085004 | Metatarsalgia | FOT |
| 102556003 | Pain in upper limb | ARM |
| 102570003 | Inguinal pain | HIP/LEG |
| 103014001 | Cervical nerve root pain | NCK |
| 10380004 | Crushing injury of finger | FTB |
| 105606008 | Injury of musculoskeletal system | UNS |
| 10601006 | Pain in lower limb | LEG |
| 1088071000000108 | Pain in upper arm | ARM |
| 11049006 | Cervical radiculitis | NCK |
| 111222003 | Derangement of medial meniscus | KNE |
| 111243002 | Bursitis of knee | KNE |
| 111640008 | Closed fracture of radius | ARM |
| 1126007 | Knee locking | KNE |
| 11654001 | Achilles tendinitis | ANK/LEG |
| 118952005 | Joint finding | UNS |
| 122481008 | Hammer toe | TOE |
| 123536004 | Sprain of upper extremity | ARM/SHD |
| 12519004 | Sprain of sacroiliac ligament | BCK/HIP |
| 125594001 | Injury of shoulder region | SHD |
| 125596004 | Injury of elbow | ELB |
| 125597008 | Injury of forearm | ARM |
| 125598003 | Injury of wrist | WRT |
| 125599006 | Injury of hand | HND |
| 125600009 | Injury of hip region | HIP |
| 125601008 | Injury of knee | KNE |
| 125602001 | Injury of lower leg | LEG |
| 125603006 | Injury of ankle | ANK |
| 125604000 | Injury of foot | FOT |
| 125605004 | Fracture of bone | UNS |
| 125606003 | Fracture of cervical spine | NCK |
| 125607007 | Fracture of thoracic spine | TXS |
| 125608002 | Fracture of lumbar spine | BCK |
| 12584003 | Bone pain | UNS |
| 12676007 | Fracture of radius | WRT/ARM |
| 127278005 | Injury of upper extremity | ARM |
| 127279002 | Injury of lower extremity | HIP |
| 129179000 | Piriformis syndrome | HIP/BCK |
| 134407002 | Chronic back pain | BCK |
| 135897006 | Salter-Harris type II | UNS |
| 13695006 | Fracture of pubic rami | HIP |
| 1539003 | Acquired trigger finger | FTB |
| 157265008 | Dislocation of hip joint | HIP |
| 157266009 | Dislocation of knee joint | KNE |
| 16114001 | Fracture of ankle | ANK |
| 161880003 | Stiff neck symptom | NCK |
| 161882006 | Stiff neck | NCK |
| 161891005 | Backache | BCK |
| 161892003 | Backache with radiation | BCK |
| 161894002 | Complaining of low back pain | BCK |
| 161896000 | Complaining of upper back ache | TXS/BCK |
| 162411004 | Complaining of stiffness | UNS |
| 162429009 | Symptom: upper limb | ARM |
| 16250001000004107 | Fracture of shoulder | SHD |
| 164539000 | O/E - joint movement painful | UNS |
| 17059001 | Prepatellar bursitis | KNE |
| 17222009 | Closed fracture of distal end of radius | WRT |
| 17883008 | Sprain of hip | HIP |
| 18171007 | Fracture of phalanx of finger | FTB |
| 18347007 | Spinal stenosis of lumbar region | BCK |
| 18876004 | Pain in finger | FTB |
| 19660004 | Disorder of soft tissue | UNS |
| 201819000 | Degenerative joint disease involving multiple joints | MSW |
| 201837004 | Localized primary osteoarthritis of the ankle and/or foot | ANK/FOT |
| 202031002 | Generalised arthritis | MSW |
| 202246002 | Recurrent dislocation of the patellofemoral joint | KNE |
| 202336002 | Acetabular labrum tear | SHD |
| 202381003 | Knee joint effusion | KNE |
| 202472008 | Hand joint pain | HND |
| 202479004 | Acromioclavicular joint pain | SHD |
| 202480001 | Elbow joint pain | ELB |
| 202482009 | Wrist joint pain | WRT |
| 202487003 | Sacroiliac joint pain | BCK/HIP |
| 202490009 | Ankle joint pain | ANK |
| 202596000 | Clicking joint | UNS |
| 202606004 | Clicking hip | HIP |
| 202609006 | Clicking knee | KNE |
| 202664003 | Cervical myelopathy | NCK |
| 202683005 | Cervical spondylosis with radiculopathy | NCK |
| 202693003 | Lumbosacral spondylosis with radiculopathy | BCK |
| 202708005 | Prolapsed lumbar intervertebral disc | BCK |
| 202732003 | Disc prolapse with radiculopathy | SPN |
| 202733008 | Cervical disc prolapse with radiculopathy | NCK |
| 202735001 | Lumbar disc prolapse with radiculopathy | BCK |
| 202752002 | Lumbar discitis | BCK |
| 202757008 | Cervical disc disorder with radiculopathy | NCK |
| 202788007 | Degenerative lumbar spinal stenosis | BCK |
| 202794004 | Lumbago with sciatica | BCK |
| 202805003 | Sacroiliac disorder | BCK/HIP |
| 202841003 | Supraspinatus tendinitis | SHD |
| 202842005 | Partial thickness rotator cuff tear | SHD |
| 202843000 | Full thickness rotator cuff tear | SHD |
| 202849001 | Subacromial impingement | SHD |
| 202855006 | Lateral epicondylitis | ELB |
| 202856007 | Biceps tendinitis | ARM |
| 202863007 | Adductor tendinitis | HIP |
| 202881005 | Tibialis posterior tendinitis | LEG |
| 202882003 | Plantar fasciitis | FOT |
| 202888004 | Anterior shin splints | LEG |
| 202900007 | Synovitis and tenosynovitis | UNS |
| 202914007 | Extensor tenosynovitis of wrist | WRT |
| 202916009 | Extensor tenosynovitis of thumb | FTB |
| 202936005 | Ganglion and cyst of synovium, tendon and bursa | UNS |
| 202942009 | Ganglion of wrist | WRT |
| 203045001 | Dupuytren's disease of palm | HND |
| 203082005 | Fibromyalgia | MSW |
| 203095000 | Spasm of back muscles | BCK |
| 203131005 | Tender heel pad | FOT |
| 203509009 | Clavicle pain | SHD |
| 203534009 | Acquired pes planus | FOT |
| 203601000 | Acquired unequal leg length | LEG |
| 203638000 | Kyphoscoliosis and scoliosis | TXS/BCK |
| 203639008 | Idiopathic scoliosis | BCK |
| 203645000 | Postural scoliosis | BCK |
| 203681002 | Acquired spondylolisthesis | BCK |
| 20511007 | Fracture of hand | HND |
| 207940009 | Closed fracture thoracic vertebra wedge | TXS |
| 207957008 | Closed fracture lumbar vertebra | BCK |
| 207959006 | Closed fracture lumbar vertebra, wedge | BCK |
| 208145002 | Fracture or disruption of pelvis | HIP |
| 208165009 | Closed fracture pelvis single pubic ramus | HIP |
| 208166005 | Closed fracture pelvis multiple pubic rami - stable | HIP |
| 208240004 | Closed fracture proximal humerus, neck | ARM/SHD |
| 208242007 | Closed fracture proximal humerus, greater tuberosity | ARM |
| 208294009 | Closed fracture olecranon, extra-articular | ELB |
| 208325003 | Closed fracture radial styloid | WRT |
| 208388003 | Fracture at wrist and/or hand level | WRT/HND |
| 208393000 | Fracture of metacarpal bone | WRT/HND |
| 208394006 | Closed fracture of metacarpal bone | HND |
| 208399001 | Closed fracture finger metacarpal neck | HND |
| 208401007 | Closed fracture finger metacarpal | HND |
| 208430000 | Closed fracture of one or more phalanges of hand | FTB |
| 208444006 | Closed fracture finger proximal phalanx | FTB |
| 208450001 | Closed fracture finger middle phalanx | FTB |
| 208634001 | Closed fracture distal tibia | LEG |
| 208687001 | Closed fracture metatarsal base | FOT |
| 208710000 | Closed fracture proximal phalanx, toe | TOE |
| 208712008 | Closed fracture distal phalanx, toe | TOE |
| 208719004 | Fracture of great toe | TOE |
| 2089002 | Paget's disease of bone | UNS |
| 208916003 | Acute meniscal tear medial posterior horn | KNE |
| 208921000 | Acute meniscal tear lateral | KNE |
| 209238002 | Closed fracture dislocation shoulder joint | SHD |
| 209354002 | Closed fracture dislocation foot | FOT |
| 209409002 | Sprains and strains of joints and adjacent muscles | UNS |
| 209436000 | Sprain of wrist and/or hand | WRT/HND |
| 209520004 | Partial tear knee anterior cruciate ligament | KNE |
| 209529003 | Sprain of ankle and/or foot | ANK/FOT |
| 209532000 | Sprain, ankle joint, lateral | ANK |
| 209557005 | Neck sprain | NCK |
| 209565008 | Lumbar sprain | BCK |
| 209574005 | Pulled back muscle | BCK |
| 209629006 | Complete tear knee anterior cruciate ligament | KNE |
| 209812006 | Sprain, symphysis pubis | HIP |
| 21351003 | Fracture of phalanx of foot | TOE |
| 21698002 | Open fracture of phalanx of finger | FTB |
| 21794005 | Radial styloid tenosynovitis | WRT |
| 221695002 | Achilles bursitis | ANK/FOT |
| 22193007 | Degenerative joint disease of hand | HND |
| 228158008 | Difficulty in walking | UNS |
| 22817005 | Strain of Achilles tendon | ANK/LEG |
| 22878006 | Contusion of knee | KNE |
| 23056005 | Sciatica | BCK/LEG |
| 23382007 | Stress fracture | UNS |
| 23406007 | Fracture of upper limb | ARM |
| 23482006 | Avulsion fracture | UNS |
| 235231000119100 | Osteophyte of bone | UNS |
| 23680005 | Enthesopathy | UNS |
| 239720000 | Tear of meniscus of knee | KNE |
| 239732001 | Disorder of patellofemoral joint | KNE |
| 239733006 | Anterior knee pain | KNE |
| 239863005 | Osteoarthritis of spinal facet joint | SPN |
| 239865003 | Osteoarthritis of acromioclavicular joint | SHD |
| 239866002 | Osteoarthritis of elbow | ELB |
| 239867006 | Osteoarthritis of wrist | WRT |
| 239868001 | Osteoarthritis of finger joint | FTB |
| 239872002 | Osteoarthritis of hip | HIP |
| 239873007 | Osteoarthritis of knee | KNE |
| 239874001 | Osteoarthritis of ankle | ANK |
| 239877008 | Osteoarthritis of first metatarsophalangeal joint | FOT/TOE |
| 239878003 | Osteoarthritis of toe joint | TOE |
| 239880009 | Lumbar spondylosis | BCK |
| 239960007 | Impingement syndrome of shoulder | SHD |
| 239961006 | Bursitis of shoulder | SHD |
| 240003004 | Suprapatellar bursitis | KNE |
| 240008008 | Synovial cyst of knee | KNE |
| 240131006 | Rhabdomyolysis | UNS |
| 240203005 | Rupture of Baker's cyst - knee | KNE |
| 240205003 | Synovial cyst | UNS |
| 240261009 | Generalised benign joint hypermobility | MSW |
| 240631000000102 | Suspected fracture or dislocation | UNS |
| 24424003 | Closed fracture of phalanx of finger | FTB |
| 24693007 | Myofascial pain syndrome | MSW |
| 247366003 | Acute back pain with sciatica | BCK |
| 247369005 | Facet joint pain | SPN |
| 247373008 | Ankle pain | ANK |
| 248491001 | Swollen knee | KNE |
| 250082003 | Hand cramps | HND |
| 250102002 | Unstable knee | KNE |
| 25415003 | Closed fracture of femur | LEG |
| 25899002 | Closed bimalleolar fracture | ANK |
| 262520005 | Thumb injury | FTB |
| 262965006 | Strain of back muscle | BCK |
| 262971000 | Tendon injury - hand | HND |
| 262981001 | Rupture of gastrocnemius tendon | LEG |
| 262992000 | Hamstring sprain | LEG |
| 263021005 | Anterior dislocation of shoulder joint | SHD |
| 263051004 | Subluxation of shoulder joint | SHD |
| 263054007 | Subluxation of finger | FTB |
| 263055008 | Subluxation of thumb | FTB |
| 263058005 | Subluxation of knee joint | KNE |
| 263084004 | Fracture dislocation of finger or thumb | FTB |
| 263128001 | Sprain of ligament of elbow | ELB |
| 263129009 | Sprain of ligament of finger | FTB |
| 263130004 | Sprain of ligament of thumb | FTB |
| 263133002 | Sprain of lateral ligament of ankle joint | ANK |
| 263199001 | Fracture of distal end of radius | ARM/WRT |
| 263225007 | Fracture of proximal end of femur | HIP |
| 263233008 | Closed fracture of femur, distal end | LEG/KNE |
| 263244000 | Bimalleolar fracture of ankle | ANK |
| 263246003 | Fracture of talus | FOT |
| 263247007 | Fracture of calcaneus | FOT |
| 263251009 | Metatarsal bone fracture | FOT |
| 26538006 | Degeneration of lumbar intervertebral disc | BCK |
| 267039000 | Swollen ankle | ANK |
| 267109007 | Symptom of ankle or foot | ANK/FOT |
| 267889007 | Generalised osteoarthritis of the hand | HND |
| 267949000 | Shoulder joint pain | SHD |
| 267953003 | Arthralgia of the lower leg | LEG |
| 267954009 | Arthralgia of the ankle and/or foot | ANK/FOT |
| 267970006 | Cervical spondylosis without myelopathy | NCK |
| 267981009 | Pain in thoracic spine | TXS |
| 267982002 | Pain in lumbar spine | BCK |
| 269062008 | Closed fracture of cervical spine | NCK |
| 269080004 | Closed fracture of lower end of humerus | ARM/ELB |
| 269083002 | Closed Colles' fracture | WRT |
| 269105005 | Dislocation or subluxation of shoulder | SHD |
| 269111008 | Dislocation or subluxation of finger or thumb | FTB |
| 269112001 | Dislocation or subluxation of knee | KNE |
| 269113006 | Acute meniscal tear medial | KNE |
| 270476009 | Wry neck/torticollis | NCK |
| 270887007 | Rupture of popliteal space synovial cyst | KNE |
| 271587009 | Stiffness | UNS |
| 271771009 | Joint swelling | UNS |
| 27182002 | Sprain of acromioclavicular ligament | SHD |
| 272009001 | Complaining of a back symptom | BCK |
| 272014002 | Complaining of foot symptom | FOT |
| 2733002 | Heel pain | FOT |
| 274142002 | Dupuytren's contracture | HND |
| 274160002 | Fracture of phalanx of thumb | FTB |
| 274162005 | Thoracic back sprain | TXS |
| 274179004 | Traumatic haematoma | UNS |
| 2764000 | Joint crepitus | UNS |
| 27741009 | Calcific tendinitis of shoulder | SHD |
| 277890004 | Swollen toe | TOE |
| 278860009 | Chronic low back pain | BCK |
| 278862001 | Acute low back pain | BCK |
| 279029001 | Pain in cervical spine | NCK |
| 279035001 | Acute thoracic back pain | BCK |
| 279038004 | Thoracic back pain | TXS |
| 279039007 | Low back pain | BCK |
| 279040009 | Mechanical low back pain | BCK |
| 279043006 | Pain in buttock | HIP/BCK |
| 279069000 | Musculoskeletal pain | UNS |
| 281531008 | Fracture of medial malleolus | KNE |
| 281535004 | Fracture of lateral malleolus | ANK |
| 281543009 | Strain of tendon of medial thigh muscle | HIP/LEG |
| 281598004 | Sprain of spinal ligament | BCK |
| 281792000 | Swollen lower leg | LEG |
| 281974002 | Weber type B fracture | ANK |
| 281975001 | Weber type A fracture | ANK |
| 282026002 | Soft tissue injury | UNS |
| 282766005 | Lower back injury | BCK |
| 282775007 | Calf injury | LEG |
| 282776008 | Injury of toe | TOE |
| 283858004 | Crush injury of thumb | FTB |
| 285365001 | Pain in toe | TOE |
| 285395009 | Strain of calf muscle | LEG |
| 29210001 | Trochanteric tendinitis | HIP |
| 297142003 | Swollen foot | FOT |
| 297193007 | Ganglion of hand | HND |
| 297194001 | Ganglion of foot | FOT |
| 298382003 | Scoliosis deformity of spine | BCK |
| 298494008 | Scoliosis of thoracic spine | TXS |
| 298857005 | Shoulder joint painful on movement | SHD |
| 299037003 | Swollen hand | HND |
| 299060006 | Swelling of finger | FTB |
| 299331007 | Bandy legged | LEG |
| 30085007 | Morton metatarsalgia | TOE |
| 300954003 | Pain in calf | LEG |
| 300955002 | Pain in thumb | FTB |
| 301813003 | Irritable hip | HIP |
| 302222008 | Elbow fracture - closed | ELB |
| 302941001 | Nonunion of fracture | UNS |
| 30556007 | Recurrent dislocation of shoulder region | SHD |
| 30760008 | Finger clubbing | FTB |
| 308153009 | Closed fracture of distal fibula | ANK/LEG |
| 309246000 | Osteoarthritis of foot joint | FOT |
| 309567004 | Toe problem | TOE |
| 30989003 | Knee pain | KNE |
| 310483003 | Complaining of pain in toe | TOE |
| 310484009 | Complaining of pain in hallux | TOE |
| 311804006 | Prolapsed lumbar intervertebral disc with sciatica | BCK |
| 311821002 | Closed fracture of great toe | TOE |
| 312225001 | Musculoskeletal and connective tissue diseases | UNS |
| 314916002 | Swollen thumb | FTB |
| 31975004 | Fracture of navicular bone of wrist | WRT |
| 31978002 | Fracture of tibia | LEG |
| 3199001 | Sprain of shoulder | SHD |
| 33173003 | Closed fracture of clavicle | SHD |
| 33192001 | Closed fracture of lower end of radius AND ulna | ARM/WRT |
| 33308003 | Disorder of back | BCK |
| 342070009 | Closed fracture of foot | FOT |
| 34268009 | Closed fracture of lateral malleolus | ANK |
| 34789001 | Pain in the coccyx | BCK |
| 34840004 | Tendinitis | UNS |
| 35678005 | Multiple joint pain | MSW |
| 359532006 | Rotator cuff impingement syndrome | SHD |
| 359817006 | Closed fracture of hip | HIP |
| 359820003 | Closed fracture of neck of femur | HIP |
| 360450007 | Strain of neck muscle | NCK |
| 36071006 | Chondromalacia of patella | KNE |
| 36186002 | Polyarthropathy | MSW |
| 36427004 | Intervertebral disc disorder | SPN |
| 36924003 | Closed fracture of metatarsal bone | FOT |
| 371081002 | Arthritis of knee | KNE |
| 371598009 | Heberden node | FTB |
| 37785001 | Patellar tendonitis | KNE |
| 37895003 | Osteoarthrosis of the carpometacarpal joint of the thumb | FTB |
| 387800004 | Cervical spondylosis | NCK |
| 387802007 | Thoracic spondylosis | TXS |
| 396275006 | Osteoarthritis | UNS |
| 39848009 | Whiplash injury to neck | NCK |
| 398878007 | Sprain of ligament | UNS |
| 399114005 | Adhesive capsulitis of shoulder | SHD |
| 399269003 | Arthropathy | UNS |
| 399346004 | Supraspinatus tear | SHD |
| 40144003 | Morning stiffness - joint | UNS |
| 404098005 | Digital mucous cyst | FTB/TOE |
| 4046000 | Degenerative spondylolisthesis | BCK |
| 405817008 | Fracture of phalanx of hand | FTB |
| 40799003 | Subacromial bursitis | SHD |
| 4106009 | Rotator cuff syndrome | SHD |
| 413428007 | Acquired kyphosis | TXS |
| 413875004 | Closed fracture of head of humerus | SHD |
| 414293001 | Fracture of tibia AND fibula | LEG |
| 41511005 | Open fracture of distal phalanx of finger | FTB |
| 415692008 | Swelling of first metatarsophalangeal joint of hallux | TOE |
| 416189003 | Exostosis | UNS |
| 416209007 | Synovitis | UNS |
| 417076003 | Dislocation of shoulder joint | SHD |
| 417109008 | Subluxation of radial head | ARM/ELB |
| 417163006 | Traumatic AND/OR non-traumatic injury | UNS |
| 417558002 | Dislocation of elbow joint | ELB |
| 417746004 | Traumatic injury | UNS |
| 418237007 | Pain in hallux | TOE |
| 42188001 | Closed fracture of ankle | ANK |
| 423849004 | Iliotibial band friction syndrome | LEG |
| 424648000 | Closed fracture of base of fifth metatarsal bone | FOT |
| 425940002 | Olecranon bursitis | ELB |
| 42636007 | Closed fracture of upper end of humerus | ARM/SHD |
| 42786005 | Snapping thumb syndrome | FTB |
| 428151000 | Closed fracture of bone of knee joint | KNE |
| 42818005 | Closed fracture of scaphoid bone of wrist | WRT |
| 428257007 | Fracture of tibial plateau | KNE |
| 428883008 | Rupture biceps tendon | ARM |
| 429513001 | Rupture Achilles tendon | ANK/LEG |
| 4308002 | Repetitive strain injury | UNS |
| 432473000 | Femoral acetabular impingement | HIP |
| 43295006 | Closed fracture of humerus | ARM |
| 43422002 | Crushing injury of foot | FOT |
| 442048005 | Tenosynovitis of wrist | WRT |
| 442056008 | Torus fracture | UNS |
| 442085002 | Greenstick fracture | UNS |
| 442520000 | Inflammation of rotator cuff tendon | SHD |
| 443700006 | Disorder of lumbar disc | BCK |
| 443798008 | Arthritis of shoulder region joint | SHD |
| 44465007 | Sprain of ankle | ANK |
| 445008009 | Ganglion | UNS |
| 447139008 | Closed fracture of tibia | LEG |
| 447395005 | Closed fracture of fibula | LEG |
| 448355005 | Greenstick fracture of distal radius | ARM/WRT |
| 448394006 | Inflammation of joint of foot | FOT |
| 448589005 | Arthritis of hand | HND |
| 449917004 | Cramp in lower limb | LEG |
| 450521003 | Osteoarthritis of patellofemoral joint | KNE |
| 45231001 | Infrapatellar bursitis | KNE |
| 45326000 | Shoulder pain | SHD |
| 45352006 | Spasm of muscle | UNS |
| 45613006 | Contusion of lower leg | LEG |
| 46866001 | Fracture of lower limb | LEG |
| 47933007 | Foot pain | FOT |
| 481000119104 | Strain of hamstring muscle | LEG |
| 48210000 | Lumbosacral spondylosis without myelopathy | BCK |
| 48532005 | Muscle strain | UNS |
| 49218002 | Hip pain | HIP |
| 49388007 | Sprain of foot | FOT |
| 51037009 | Fracture of patella | KNE |
| 52011008 | Injury of finger | FTB |
| 53057004 | Hand pain | HND |
| 53208009 | Peroneal tendinitis | ANK/FOT |
| 53226007 | Pes planus | FOT |
| 53286005 | Medial epicondylitis of elbow joint | ELB |
| 53627009 | Closed fracture of radius AND ulna | ARM/WRT |
| 54556006 | Fracture of ulna | ARM/WRT |
| 54888009 | Sprain of knee | KNE |
| 55146009 | Sacroiliitis | BCK |
| 55260003 | Calcaneal spur | FOT |
| 55300003 | Cramp | UNS |
| 56608008 | Pain in wrist | WRT |
| 57406009 | Carpal tunnel syndrome | WRT/HND |
| 57676002 | Joint pain | UNS |
| 58150001 | Fracture of clavicle | SHD |
| 58580000 | Closed supracondylar fracture of humerus | ARM/ELB |
| 58781003 | Gluteal tendinitis | HIP |
| 5913000 | Fracture of neck of femur | HIP |
| 61007003 | Separation of symphysis pubis during delivery | HIP |
| 62629000 | Divarication of recti | ABD |
| 637091000000105 | Osteoarthritis NOS, of the lower leg | KNE |
| 64217002 | Curvature of spine | TXS/BCK |
| 64298006 | Mallet finger | FTB |
| 64455005 | Fracture of acetabulum | HIP/SHD |
| 64665009 | Closed fracture of calcaneus | FOT |
| 65260001 | Cervical spondylosis with myelopathy | NCK |
| 65358001 | Acquired hallux valgus | TOE |
| 65966004 | Fracture of forearm | ARM |
| 66308002 | Fracture of humerus | ARM |
| 6654000 | Acquired hallux rigidus | TOE |
| 6698000 | Closed trimalleolar fracture | ANK |
| 67315001 | Degenerative joint disease of shoulder region | SHD |
| 67801009 | Tenosynovitis | HND |
| 68449006 | Arthritis of hip | HIP |
| 6858004 | Capsulitis | UNS |
| 68854005 | Closed fracture of head of radius | ARM/ELB |
| 68962001 | Muscle pain | UNS |
| 69195002 | Degeneration of cervical intervertebral disc | NCK |
| 699062006 | Injury of shoulder and upper arm | SHD/ARM |
| 699370008 | Symptom of foot | FOT |
| 699462004 | Monoarthritis | UNS |
| 70070008 | Torticollis | NCK |
| 704213001 | Closed fracture of phalanx of thumb | FTB |
| 70704007 | Sprain of wrist | WRT |
| 712893003 | Traumatic and/or non-traumatic injury of back | BCK |
| 71620000 | Fracture of femur | LEG |
| 72047008 | Osgood Schlatter disease | KNE |
| 721291009 | Disorder of patella, unspecified | KNE |
| 73589001 | Intervertebral disc prolapse | SPN |
| 74323005 | Pain in elbow | ELB |
| 74779009 | Strain of rotator cuff capsule | SHD |
| 74814004 | Contusion of foot | FOT |
| 75308009 | Closed fracture of navicular bone of foot | FOT |
| 75591007 | Fracture of fibula | LEG |
| 75857000 | Fracture of radius AND ulna | ARM |
| 76107001 | Spinal stenosis | BCK |
| 7674000 | Greater trochanteric pain syndrome | HIP |
| 76865005 | Closed fracture of distal phalanx of finger | FTB |
| 771083005 | Pain in upper arm | ARM |
| 77493009 | Fracture of pelvis | HIP |
| 77547008 | Degeneration of intervertebral disc | SPN |
| 78435003 | Ganglion of joint | UNS |
| 78514002 | Thigh pain | LEG |
| 788465007 | Repetitive motion disorder | UNS |
| 789758005 | Strain of rotator cuff of shoulder | SHD |
| 80068009 | Swelling of limb | LEG/ARM |
| 80692000 | Late effect of tendon injury | UNS |
| 80756009 | Closed fracture of patella | KNE |
| 81498004 | Bursitis of hip | HIP |
| 81576005 | Closed fracture of phalanx of foot | FOT/TOE |
| 81680005 | Neck pain | NCK |
| 81902001 | Sprain of medial collateral ligament of knee | KNE |
| 82423001 | Chronic pain | UNS |
| 82675004 | Baker's cyst | KNE |
| 82991003 | Generalized aches and pains | MSW |
| 83561009 | Spinal stenosis in cervical region | NCK |
| 84017003 | Bursitis | UNS |
| 84445001 | Joint stiffness | UNS |
| 84869007 | Musculoskeletal symptoms | UNS |
| 85551004 | Hypermobility syndrome | MSW |
| 86380000 | Acquired claw toes | TOE |
| 870206000 | Weber type A fracture | ANK |
| 870207009 | Weber type B fracture | ANK |
| 87778004 | Sprain of hand | HND |
| 8847002 | Spondylosis | SPN |
| 88998003 | Osteophyte | UNS |
| 90460009 | Injury of neck | NCK |
| 906591000006102 | Soft tissue injuries | UNS |
| 90834002 | Pain in limb | ARM |
| 91037003 | Closed fracture of pelvis | HIP |
| 926335004 | Rupture of rotator cuff of shoulder | SHD |
| 928000 | Disorder of musculoskeletal system | UNS |
| 939761000006103 | Musculoskeletal pain mild | UNS |
| 9468002 | Closed fracture of carpal bone | WRT/HND |
| 95414005 | Calcific tendinitis | UNS |
| 95854004 | Pulled elbow | ELB |
| 9682006 | Fracture of scapula | SHD |
| 9808005 | Closed fracture of cuboid bone of foot | FOT |
| 1931871000006102 | Suspected fracture | UNS |
| **ABD** Abdomen; **ANK** Ankle; **ARM** Arm; **BCK** Back/lumbar spine; **CHE** Chest; **ELB** Elbow; **FOT** Foot; **FTB** Finger(s)/thumb; **HIP** Hip/pelvis; **HND** Hand; **KNE** Knee; **LEG** Leg; **MSW** Multiple-site/widespread; **NCK** Neck/cervical spine; **SHD** Shoulder; **SKF** Skull/head/face; **SPN** Spine; **TNK** Trunk, **TOE** Toe; **TXS** Thoracic spine; **UNS** Unspecified body region; **WRT** Wrist | | |

Inflammatory exclusions codelist

1. **List of covariates and sources**

| **Covariate** | **Source** |
| --- | --- |
| **Registered population characteristics** | |
| Female (%) | NHS Digital. Patients registered at a GP Practice, Nov 2021-Jun 2022 |
| Aged 65+ years (%) | NHS Digital. Patients registered at a GP Practice, Nov 2021-Jun 2022 |
| Aged 85+ years (%) | NHS Digital. Patients registered at a GP Practice, Nov 2021-Jun 2022 |
| Black, Asian, Minority Ethnic background (%) | OHID. National General Practice Profiles |
| Practice weighted deprivation decile (1-10)† | OHID. National General Practice Profiles |
| **Practice organisation and performance characteristics** | |
| Total population size (n) | NHS Digital. Patients registered at a GP Practice, Nov 2021-Jun 2022 |
| Total clinical staff FTE per 10,000 | OHID. GP Profiles for Patients, Q4 2021-Q2 2022  NHS Digital. Primary Care Workforce - Quarterly Update; NHS Digital. General Practice Workforce; |
| QOF Overall achievement (max=635) | NHS Digital. Quality and Outcomes Framework, 2021-2022 |
| Positive experience of care (%) | GP Patient Survey, 2022 |
| CQC Overall Rating‡ | OHID. National General Practice Profiles (last available) |
| Number of selected services available/accessible to MSK patients (0-11) | Brief online questionnaire to practices (see below) |
| Number of types of MSK clinical decision support systems used (0-8) | Brief online questionnaire to practices (see below) |
| **Need-related factors** | |
| Prevalence of self-reported long-term MSK problem (%) | GP Patient Survey, 2022 |
| Prevalence of chronic pain (%) | PRELIM Survey, 2017; Lynch et al., 2023 |
| Prevalence of high-impact chronic pain (%) | PRELIM Survey, 2017; Lynch et al., 2023 |
| QOF Obesity prevalence (%) | NHS Digital. Quality and Outcomes Framework, 2021-2022 |
| **CQC** Care Quality Commission; **FTE** Full Time Equivalent; **QOF** Quality and Outcomes Framework; **OHID** Office for Health Improvement & Disparities **MSK** Musculoskeletal | |

**Questions from brief online GP practice questionnaire used in the current study**

| Which of the following options are available to your practice (or PCN) for your MSK patients? |
| --- |
| 1. Access to a social prescribing team 2. Access to specific healthy lifestyle support programmes, e.g. council run, voluntary or third sector organisations providing weight management programmes 3. The “ESCAPE Pain” knee rehabilitation programme (or an equivalent exercise-based exercise programme) 4. An annual health check to assess/review patients with long-term MSK problems (e.g. fibromyalgia) 5. Access to generic mental health services 6. Access to specific mental health care for MSK disorders such as a multidisciplinary pain clinic or psychologically informed treatment clinic for patients with complex needs 7. Vocational support, e.g. via a vocational advisor or workplace assessor 8. Access to MRI scans for patients with MSK disorders 9. Access to MSK joint injections in primary care 10. Access to Yoga for Healthy Lower Backs 11. Direct access or self-referral to physiotherapy   **Variable for analysis = simple count (0-11)** |

| Do your clinicians use any of the following MSK clinical decision support systems (typically these are digital/online systems)? |
| --- |
| 1. MSK red flag identification (e.g. software or online protocols to support local guidance and SOPs) 2. Diagnostic decision support (e.g. *Orthopathways* or *MSK Predict*) 3. MSK risk stratification (e.g. STarT Back Tool) 4. MSK prescribing support (e.g. analgesic ladder protocol available online) 5. MSK patient self-management (e.g. apps, leaflets, online support networks, or links to MSK websites) 6. MSK referral optimisation to specialist MSK services (e.g. protocols for referral to ortho, neuro, or rheumatology) 7. MSK online imaging protocols (e.g. *iRefer*) 8. MSK shared decision-making tools (e.g. knee & hip surgery decision support tool from Versus Arthritis)   **Variable for analysis = simple count (0-8)** |

1. **Technical Appendix: Method for obtaining practice-specific modelled prevalence estimates of chronic pain and high impact chronic pain**

We derived practice-specific estimates of chronic pain and high impact chronic pain in adults aged 35+ years from previously published LSOA-specific modelled estimates that were based on the PRELIM survey conducted in 2017 (source: Lynch et al., 2023). We used GP-LSOA cross-mapping from NHS Digital GP Data Hub (January 2022 extract as being closest to the mid-point of MIDAS-GP recruitment) to derive the proportion of the total practice population resident in each LSOA. We then applied our modelled LSOA-specific prevalence estimates to these to yield weighted practice-specific estimates of chronic pain and high impact chronic pain in adults aged 35+ years. This assumed that the neighbourhood distribution of 35+ year-olds was the same as the total registered population (i.e. all ages). The registered practice populations for the 30 participating practices in MIDAS-GP resided in a total of 397 LSOAs, including all 298 LSOAs in North Staffordshire & Stoke-on-Trent. LSOA was unspecified for 33 patients registered with 17 practices. The median number of LSOAs covered by each practice was 97.5 (IQR: 53.25, 150.5; range 21 – 233). Of 258,287 total population (all ages), 6795 (2.6%) resided in out-of-area neighbourhoods, most commonly LSOAs in neighbouring areas in Stafford (N=4876), Cheshire East (N=1096), and Shropshire (N=648). Modelled LSOA-specific prevalence estimates were only available for LSOAs in North Staffordshire and Stoke-on-Trent. For out-of-area and missing LSOAs, we used the median LSOA-specific prevalence estimate.

1. **Data tables and scatterplots**

**D1. Proportion of adults consulting for a MSK pain condition vs length of recruitment period.** Each marker represents a GP practice. Y-axis represents component+residual from regression analysis using fractional polynomials with days of recruitment and whether the practice was open for recruitment in December as independent variables. As expected the proportion of the registered adult population consulting is higher in practices with longer recruitment periods but there is up to two-fold variation among practices with similar lengths of recruitment period.

**
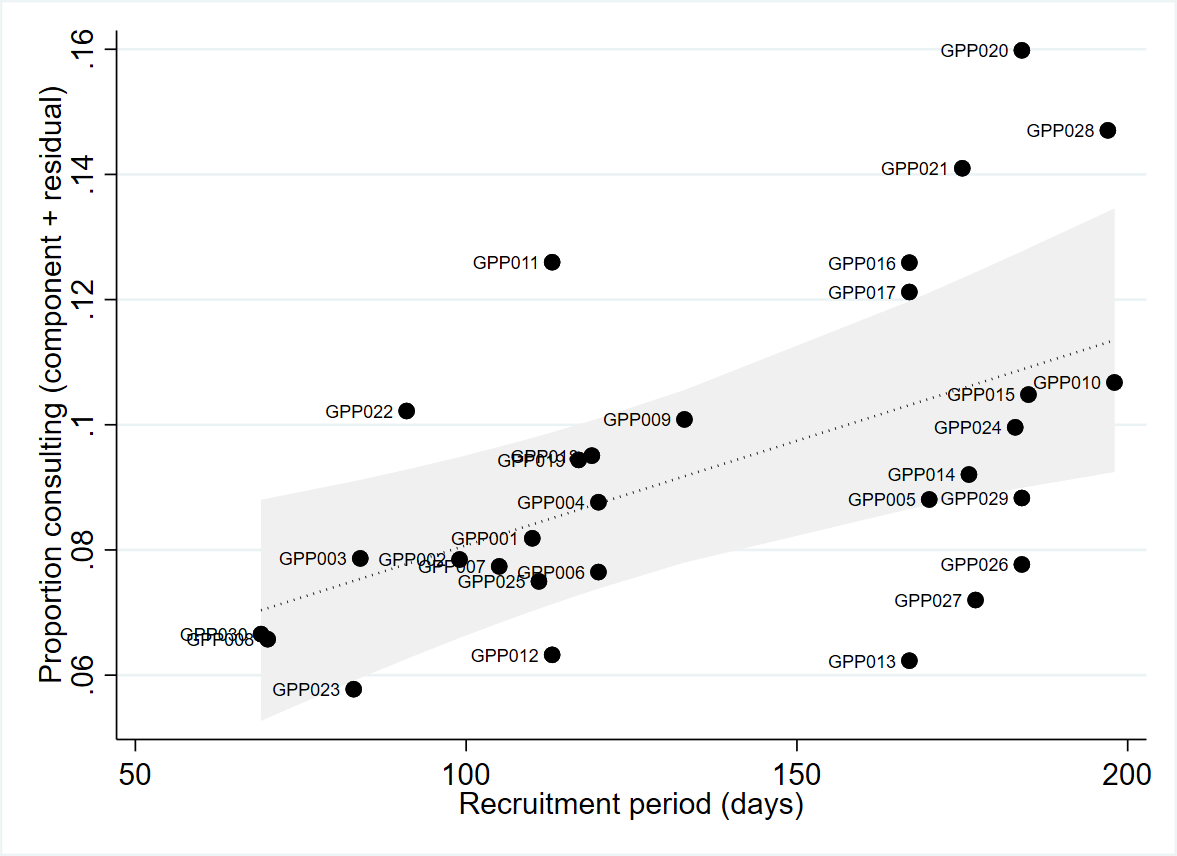
**

**D2. Proportion of adults consulting for a MSK Pain Condition, by GP practice: North Staffordshire & Stoke-on-Trent**

| **GP Practice** | **Recruitment period (days)** | **Total registered population 18+§** | **Total†** | | | **After exclusions‡** | | |
| --- | --- | --- | --- | --- | --- | --- | --- | --- |
|  |  |  | **n** | **Pr** | **95%CI** | **n** | **Pr** | **95%CI** |
| GPP020 | 184 | 7,083 | 1,127 | 0.159 | 0.151, 0.168 | 357 | 0.050 | 0.046, 0.056 |
| GPP028 | 197 | 3,007 | 440 | 0.146 | 0.134, 0.159 | 351 | 0.116 | 0.106, 0.129 |
| GPP021 | 175 | 3,646 | 514 | 0.141 | 0.130, 0.153 | 473 | 0.130 | 0.119, 0.141 |
| GPP011 | 113 | 11,105 | 1,391 | 0.125 | 0.119, 0.132 | 462 | 0.042 | 0.038, 0.045 |
| GPP016 | 167 | 3,203 | 401 | 0.125 | 0.114, 0.137 | 353 | 0.110 | 0.010, 0.122 |
| GPP017 | 167 | 2,508 | 304 | 0.121 | 0.109, 0.135 | 276 | 0.110 | 0.098, 0.123 |
| GPP010 | 198 | 6,601 | 700 | 0.106 | 0.099, 0.114 | 455 | 0.069 | 0.063, 0.075 |
| GPP015 | 185 | 4,351 | 453 | 0.104 | 0.094, 0.114 | 368 | 0.085 | 0.077, 0.093 |
| GPP022 | 91 | 11,086 | 1,133 | 0.102 | 0.097, 0.108 | 1064 | 0.096 | 0.091, 0.102 |
| GPP009 | 133 | 9,137 | 915 | 0.100 | 0.094, 0.106 | 598 | 0.065 | 0.061, 0.071 |
| GPP024 | 183 | 3,843 | 380 | 0.099 | 0.090, 0.109 | 318 | 0.083 | 0.074, 0.092 |
| GPP018 | 119 | 6,469 | 615 | 0.095 | 0.088, 0.102 | 561 | 0.087 | 0.080, 0.094 |
| GPP019 | 117 | 5,658 | 530 | 0.094 | 0.086, 0.102 | 462 | 0.082 | 0.075, 0.089 |
| GPP014 | 176 | 7,335 | 670 | 0.091 | 0.085, 0.098 | 451 | 0.061 | 0.056, 0.067 |
| GPP004 | 120 | 7,635 | 669 | 0.088 | 0.081, 0.094 | 622 | 0.081 | 0.076, 0.088 |
| GPP029 | 184 | 7,695 | 674 | 0.088 | 0.081, 0.094 | 462 | 0.060 | 0.055, 0.066 |
| GPP005 | 170 | 5,495 | 480 | 0.087 | 0.080, 0.095 | 428 | 0.078 | 0.071, 0.085 |
| GPP001 | 110 | 8,503 | 690 | 0.081 | 0.076, 0.087 | 478 | 0.056 | 0.052, 0.061 |
| GPP003 | 84 | 6,167 | 485 | 0.079 | 0.072, 0.086 | 444 | 0.072 | 0.066, 0.079 |
| GPP002 | 99 | 7,214 | 561 | 0.078 | 0.072, 0.084 | 448 | 0.062 | 0.057, 0.068 |
| GPP007 | 105 | 8,401 | 650 | 0.077 | 0.072, 0.083 | 487 | 0.058 | 0.053, 0.063 |
| GPP026 | 184 | 4,690 | 361 | 0.077 | 0.070, 0.085 | 257 | 0.055 | 0.049, 0.062 |
| GPP006 | 120 | 9,623 | 729 | 0.076 | 0.071, 0.081 | 605 | 0.063 | 0.058, 0.068 |
| GPP025 | 111 | 8,968 | 666 | 0.074 | 0.069, 0.080 | 554 | 0.062 | 0.057, 0.067 |
| GPP027 | 177 | 4,625 | 333 | 0.072 | 0.065, 0.080 | 305 | 0.066 | 0.059, 0.073 |
| GPP030 | 69 | 2,960 | 197 | 0.067 | 0.058, 0.076 | 186 | 0.063 | 0.055, 0.072 |
| GPP008 | 70 | 8,321 | 547 | 0.066 | 0.061, 0.071 | 507 | 0.061 | 0.056, 0.066 |
| GPP012 | 113 | 10,091 | 631 | 0.063 | 0.058, 0.067 | 563 | 0.056 | 0.051, 0.060 |
| GPP013 | 167 | 10,544 | 657 | 0.062 | 0.058, 0.067 | 616 | 0.058 | 0.054, 0.063 |
| GPP023 | 83 | 8,503 | 485 | 0.057 | 0.052, 0.062 | 450 | 0.053 | 0.048, 0.058 |
| Median | 126.5 | 7,149 | 588 | 0.088 |  | 459 | 0.064 |  |
| TOTAL | 4,171 | 204,467 | 18,388 | 0.090 | 0.089, 0.091 | 13,961 | 0.068 | 0.067, 0.069 |
| † Unique individuals consulting with a Keele 500 MSK Pain code during the recruitment period at a participating practice  ‡ Unique individuals consulting with a Keele 500 MSK Pain code during the recruitment period at a participating practice, excluding cases with a recorded code for inflammatory musculoskeletal disease in past 3 years  § Registered population aged 18 years and over at the practice-specific mid-point of recruitment (data source: NHS Digital ‘Patients Registered at a GP Practice’ [Patients registered at a GP practice - NHS Digital](https://digital.nhs.uk/data-and-information/data-tools-and-services/data-services/general-practice-data-hub/patients-registered-at-a-gp-practice)) | | | | | | | | |

**D3. Relationships between proportion consulting for MSK pain condition and selected covariates.**

In all figures, y-axis represents predicted + residual values for dependent variable (proportion consulting) from regression analysis using fractional polynomials for independent variables and with adjustment for length of recruitment period, whether practice recruitment period included December, proportion of registered population female, proportion of registered population aged 65+ years.

| **vs registered population female** | **vs registered population aged 65+ years** |
| --- | --- |
| **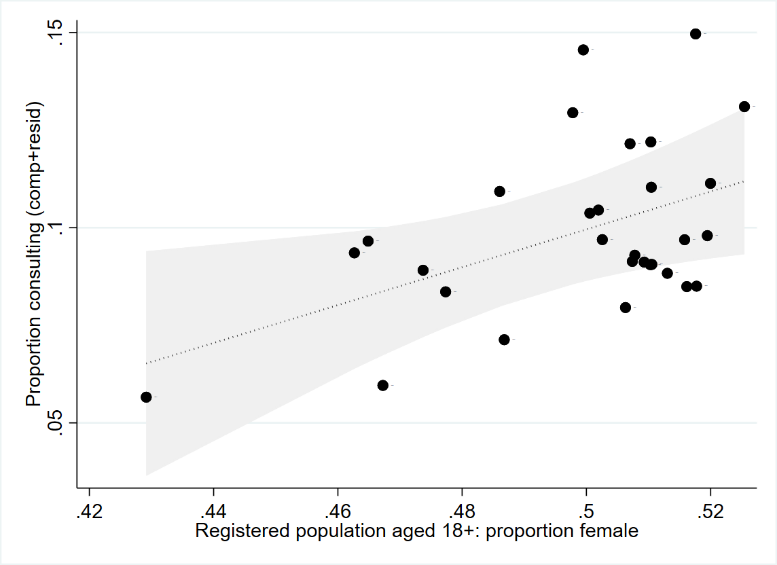** | **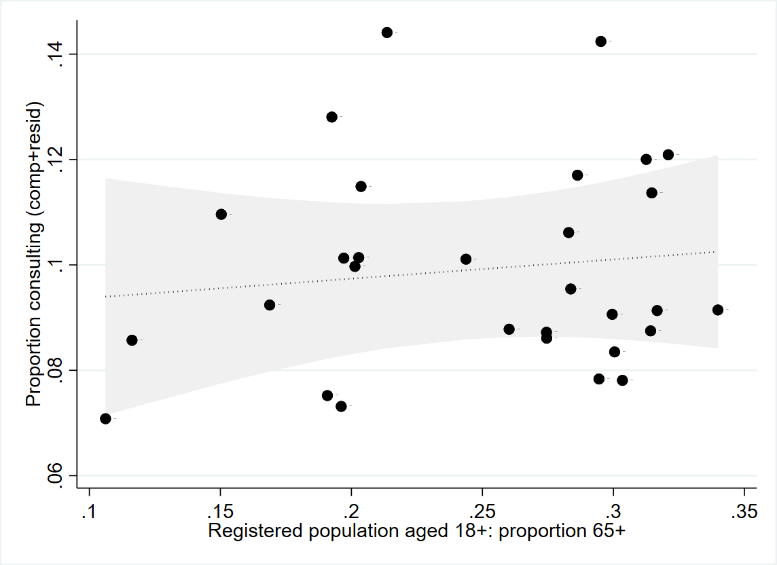** |
| LR χ^2^(1)=5.87, p=0.0154 | LR χ^2^(1)=0.35, p=0.5569 |

| **vs registered population aged 85+ years** | **vs registered population Black, Asian, or Minority Ethnic background** |
| --- | --- |
| **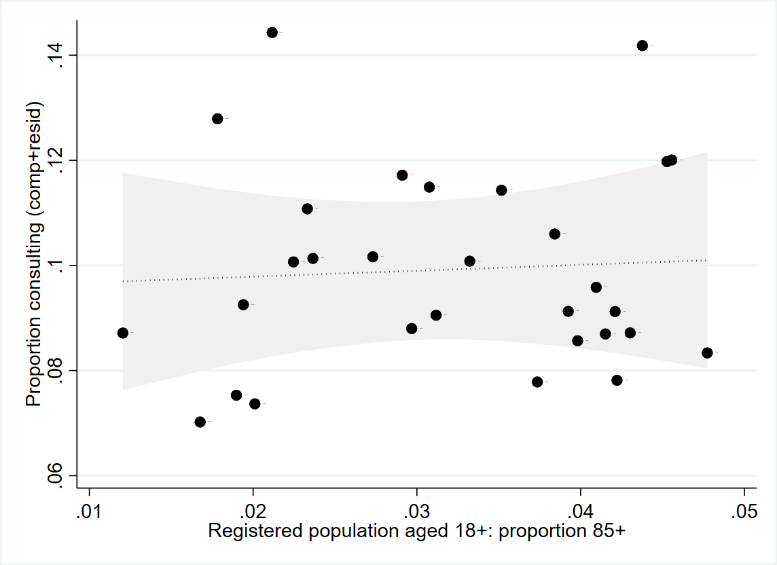** | **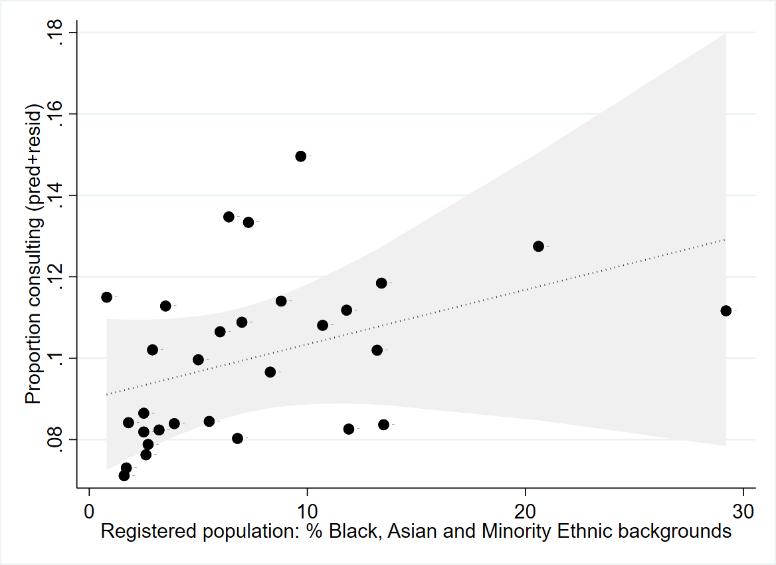** |
| LR χ^2^(1)=0.07, p=0.7860 | LR χ^2^(1)=1.75, p=0.1863 |

| **vs practice weighted Index of Multiple Deprivation (1=most deprived)** | **vs size of registered population (all ages)** |
| --- | --- |
| **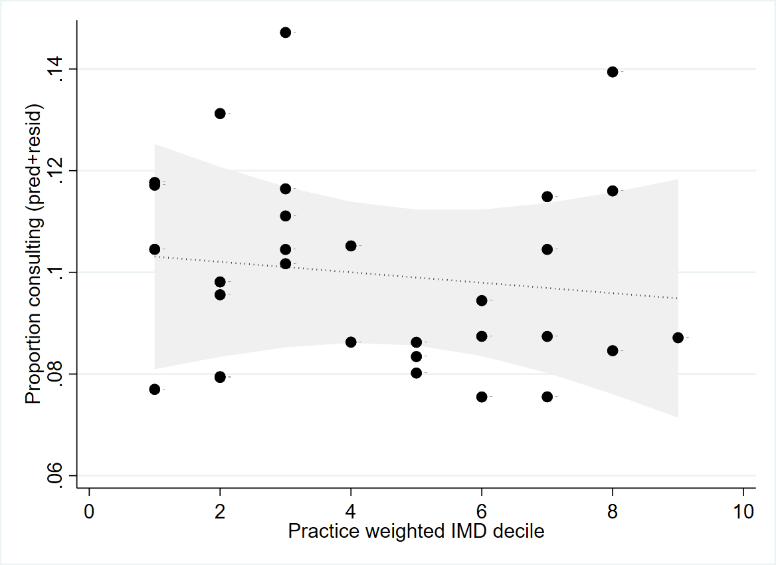** | **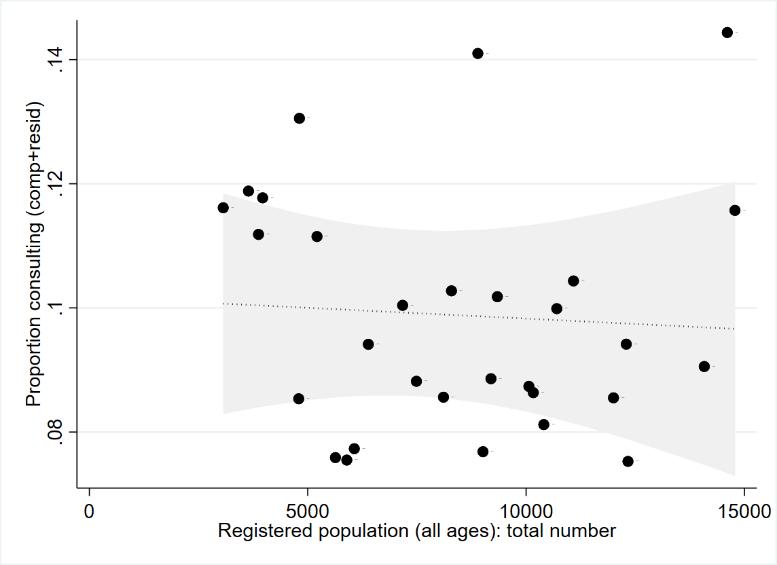** |
| LR χ^2^(1)=0.24, p=0.6261 | LR χ^2^(1)=0.79, p=0.3750 |

| **vs Clinical staff FTE per 10,000 registered patients** | **vs Overall achievement points in Quality and Outcomes Framework** |
| --- | --- |
| **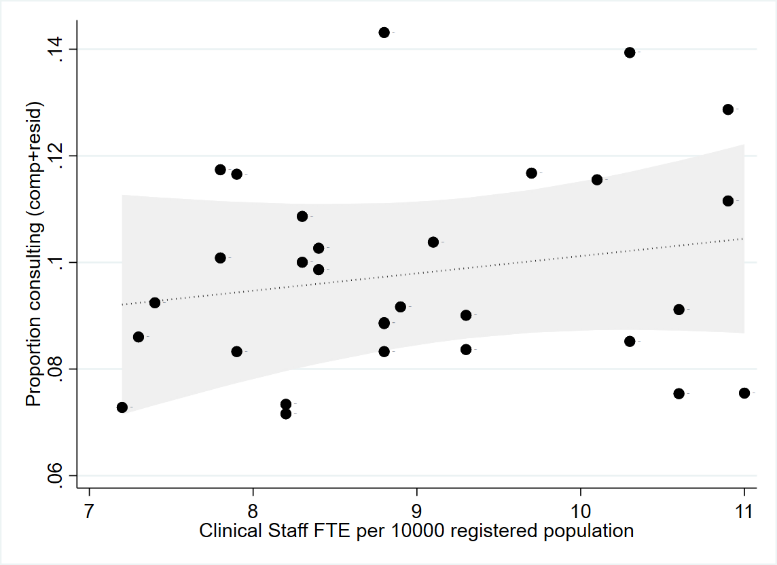** | **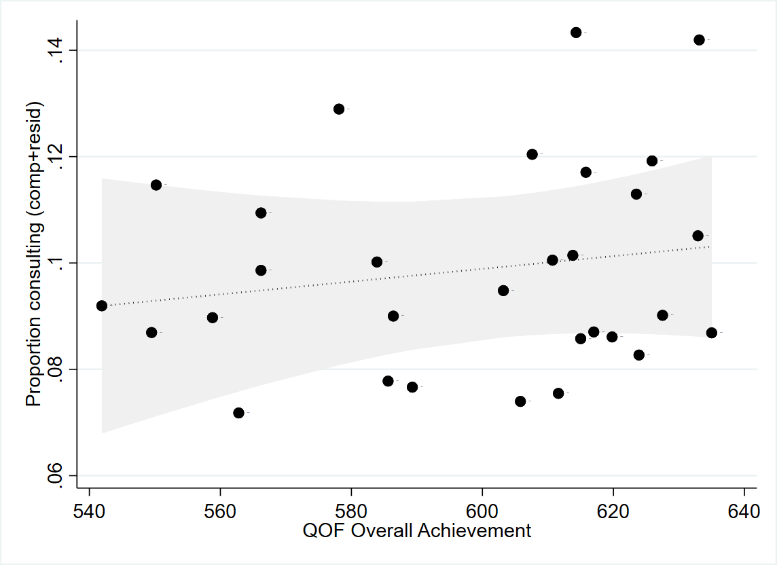** |
| LR χ^2^(1)=0.94, p=0.3316 | LR χ^2^(1)=0.62, p=0.4295 |

| **vs Percentage of patients reporting positive experience** | **vs Prevalence of obesity in adults** |
| --- | --- |
| **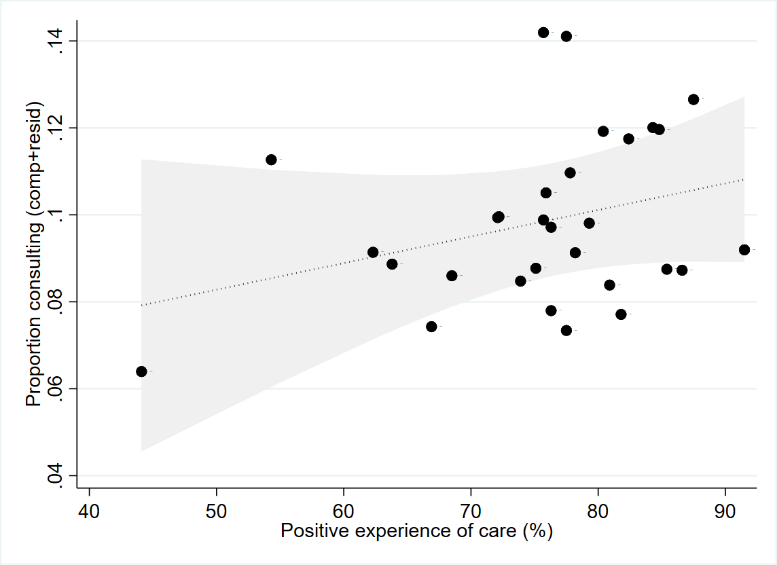** | **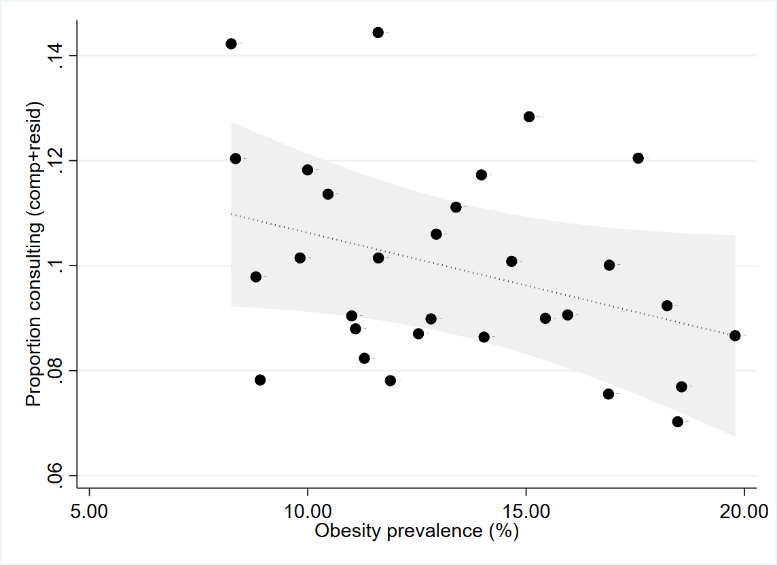** |
| LR χ^2^(1)=1.94, p=0.1641 | LR χ^2^(1)=3.45, p=0.0633 |

| **vs opioid items per 1000 patients** | **vs available services** |
| --- | --- |
| **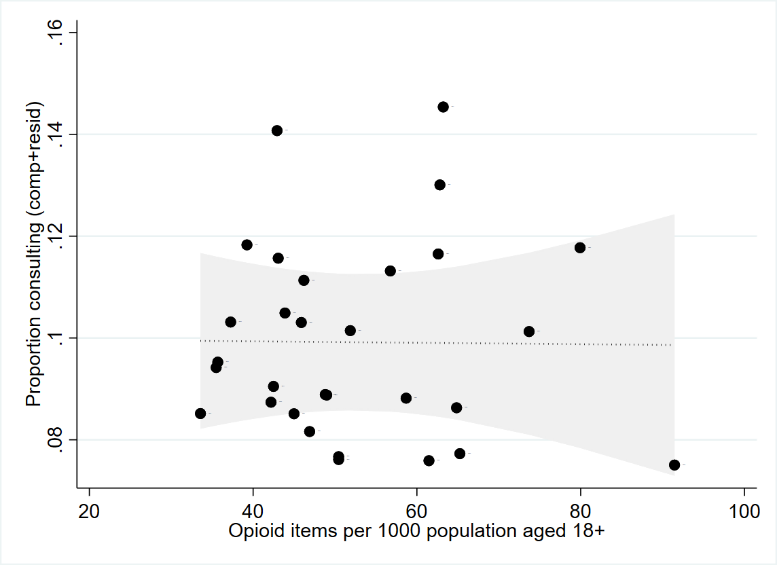** | **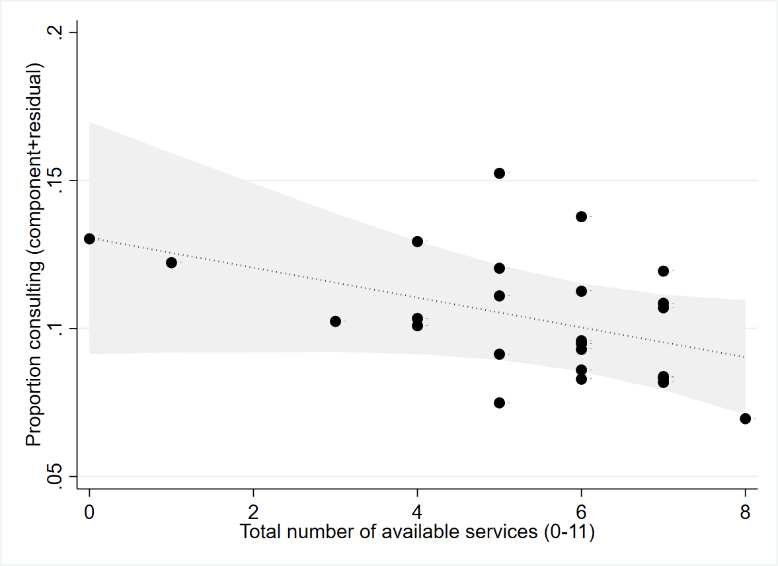** |
| LR χ^2^(1)=0.00, p=0.9564 | LR χ^2^(1)=3.25, p=0.0715 (n=25 practices) |

| **vs clinical decision support systems used** |
| --- |
| **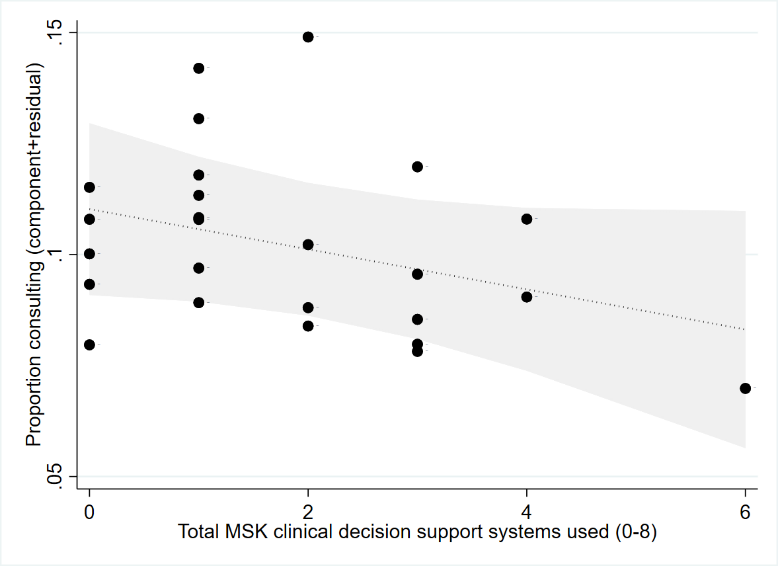** |
| LR χ^2^(1)=2.95, p=0.0860 |

**D4. Sensitivity analysis 1 – exclusion of consulting cases with a previous record of inflammatory arthritis**

In all figures, y-axis represents component + residual values for dependent variable (proportion consulting) from regression analysis using fractional polynomials for independent variables and with adjustment for length of recruitment period, whether practice recruitment period included December, proportion of registered population female, proportion of registered population aged 65+ years.

| **vs prevalence of long-term MSK problem** | **vs prevalence of chronic pain** |
| --- | --- |
| **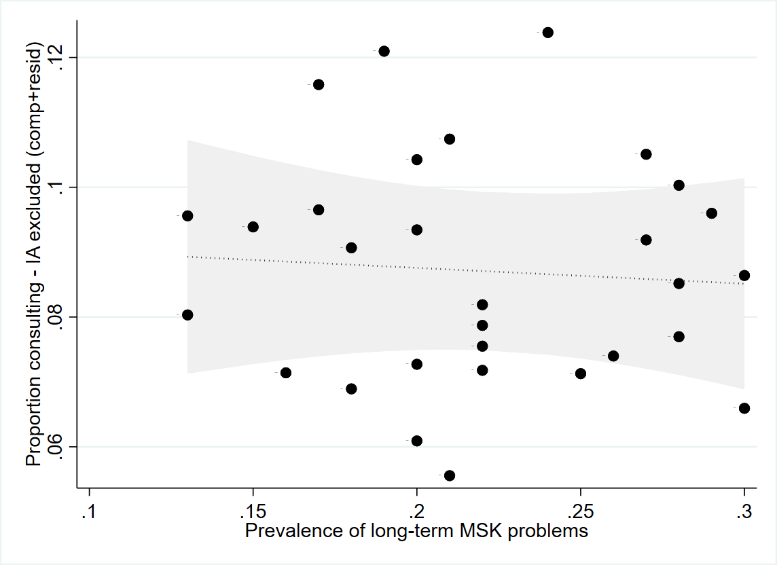** | **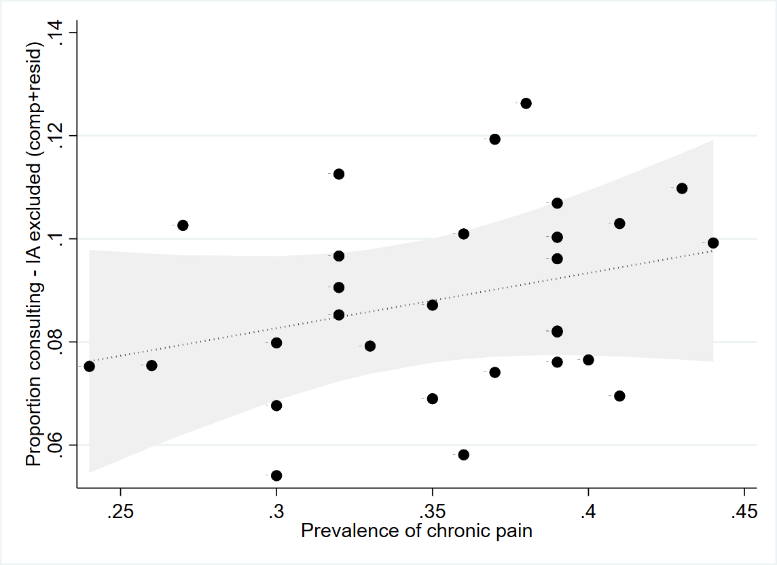** |
| LR χ^2^(1)=0.14, p=0.7052 | LR χ^2^(1)=1.66, p=0.1977 |

| **vs prevalence of high-impact chronic pain** |
| --- |
| **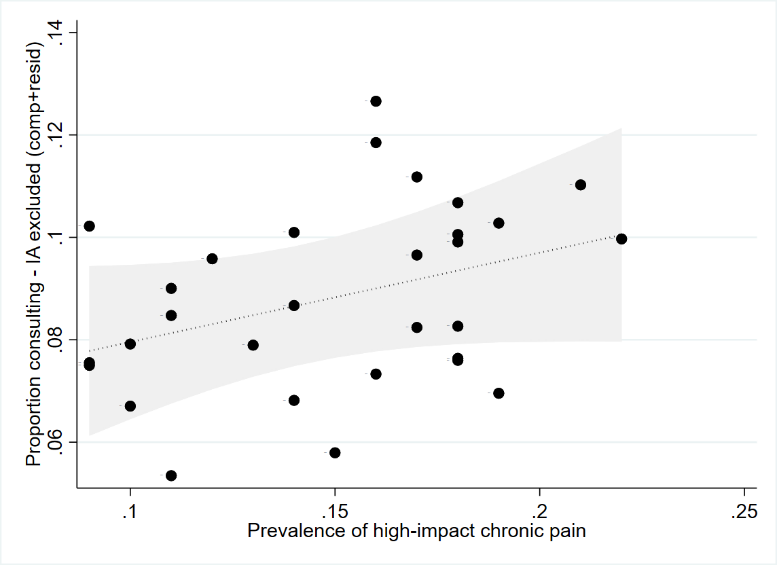** |
| LR χ^2^(1)=2.79, p=0.0949 |

| **vs registered population female** | **vs registered population aged 65+ years** |
| --- | --- |
| **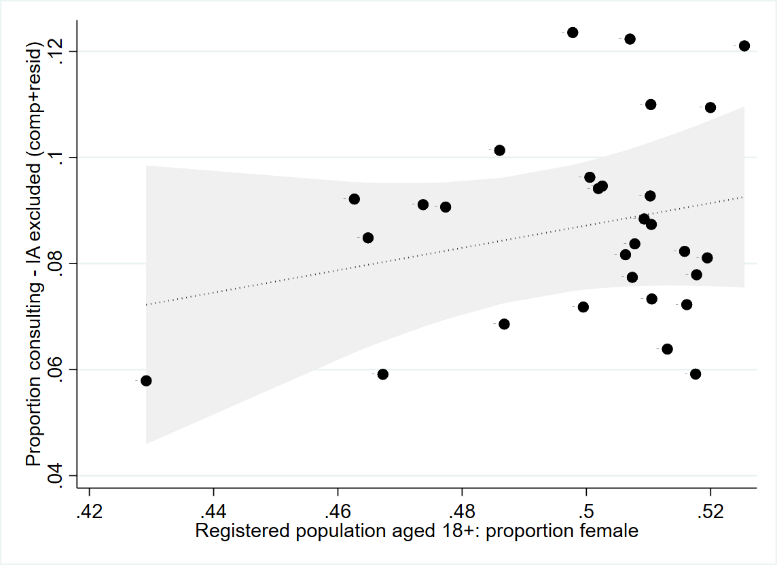** | **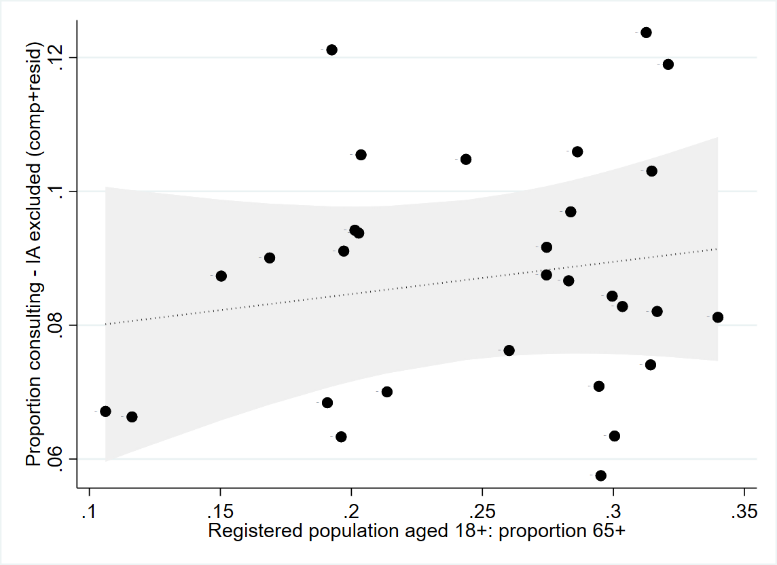** |
| LR χ^2^(1)=1.44, p=0.2305 | LR χ^2^(1)=0.71, p=0.3978 |

| **vs registered population aged 85+ years** | **vs registered population Black, Asian, or Minority Ethnic background** |
| --- | --- |
| **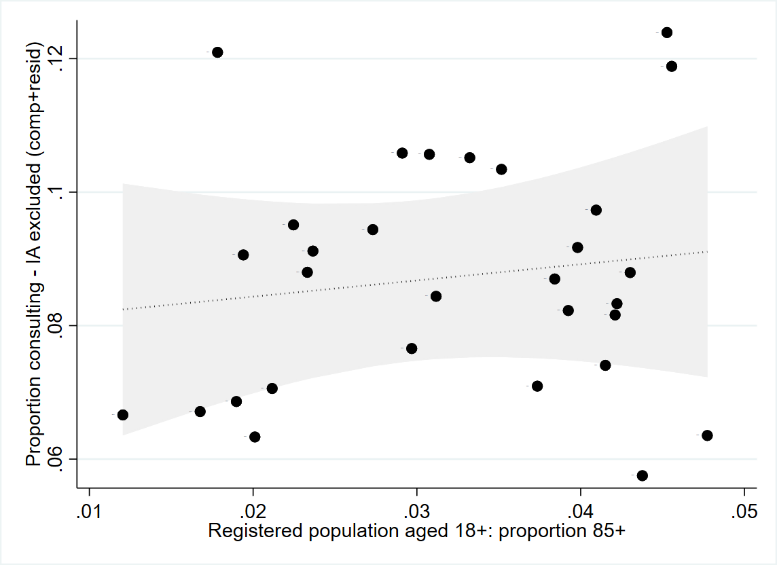** | **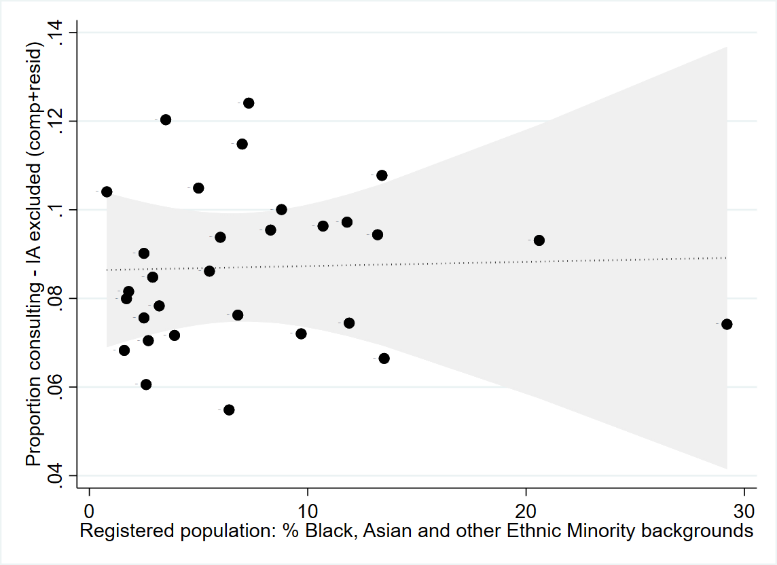** |
| LR χ^2^(1)=0.41, p=0.5229 | LR χ^2^(1)=0.01, p=0.9190 |

| **vs practice weighted Index of Multiple Deprivation (1=most deprived)** | **vs size of registered population (all ages)** |
| --- | --- |
| **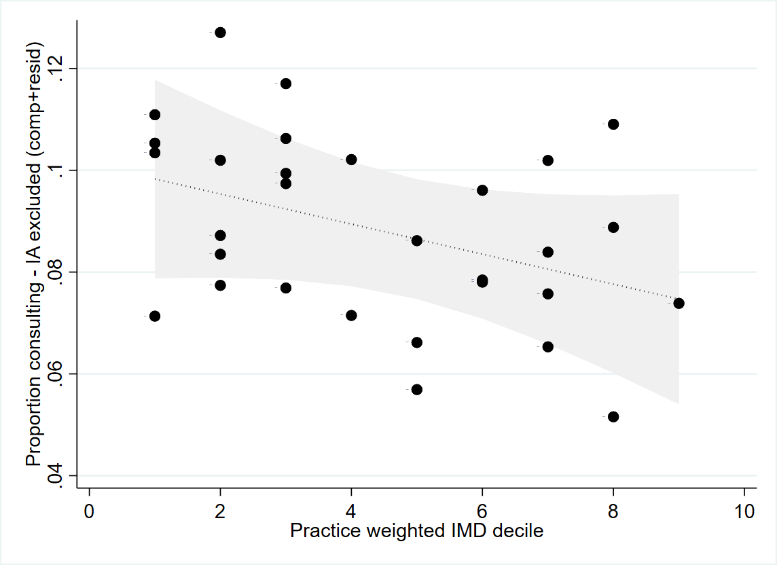** | **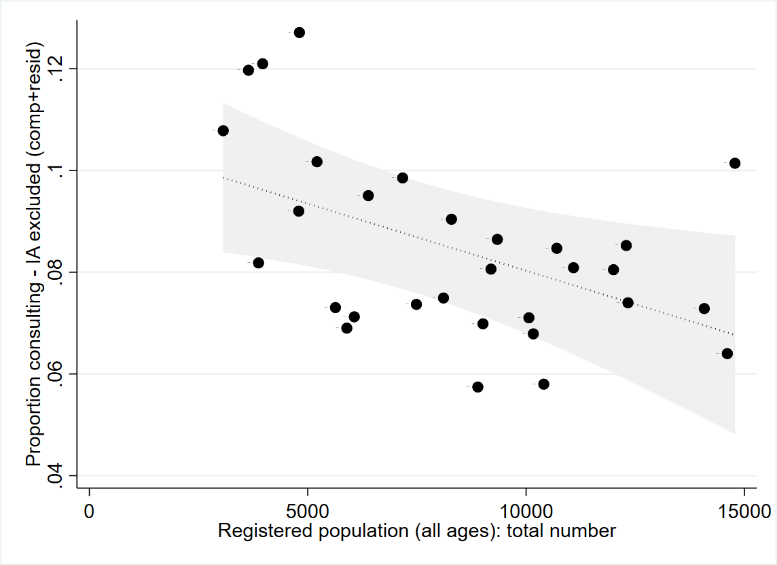** |
| LR χ^2^(1)=2.43, p=0.1188 | LR χ^2^(1)=9.05, p=0.0026 |

| **vs Clinical staff FTE per 10,000 registered patients** | **vs Overall achievement points in Quality and Outcomes Framework** |
| --- | --- |
| **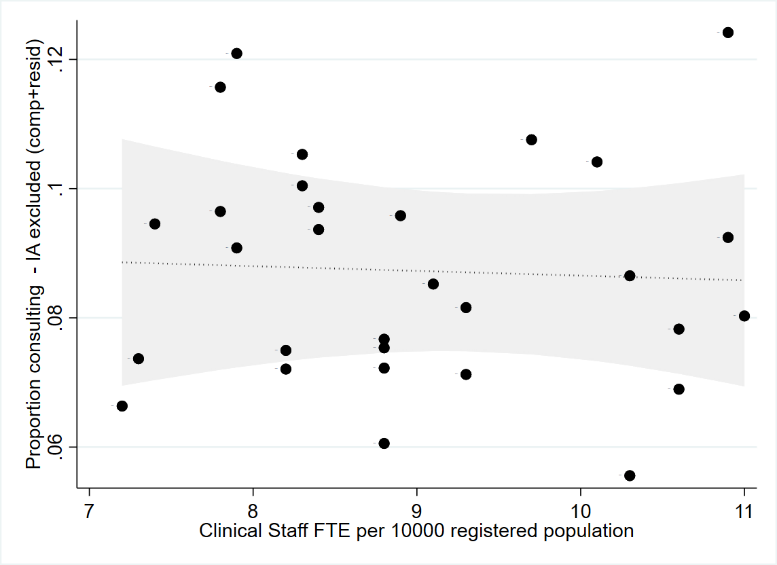** | **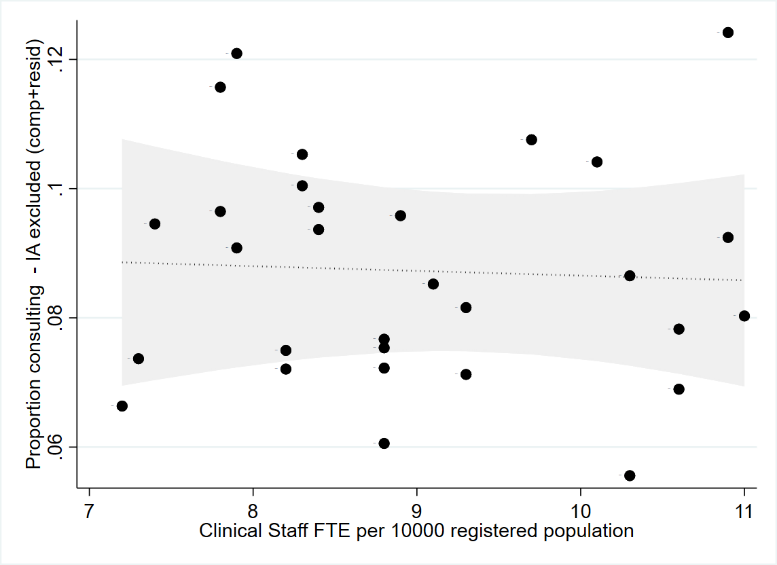** |
| LR χ^2^(1)=0.06, p=0.8119 | LR χ^2^(1)=1.13, p=0.2877 |

| **vs Percentage of patients reporting positive experience** | **vs Prevalence of obesity in adults** |
| --- | --- |
| **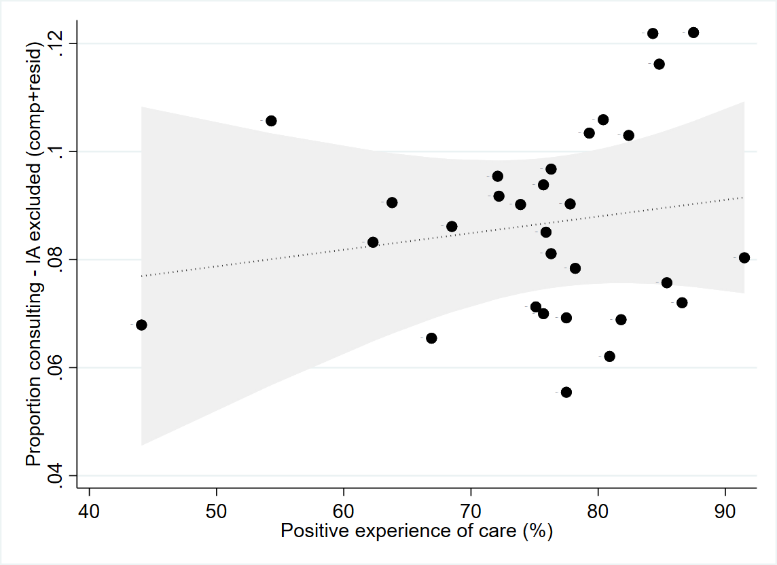** | **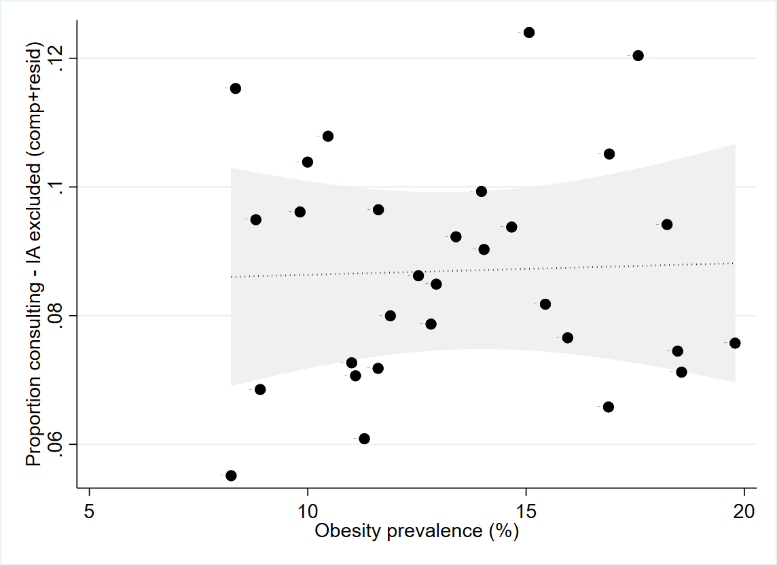** |
| LR χ^2^(1)=0.58, p=0.4480 | LR χ^2^(1)=0.03, p=0.8572 |

| **vs opioid items per 1000 patients** | **vs available services** |
| --- | --- |
| **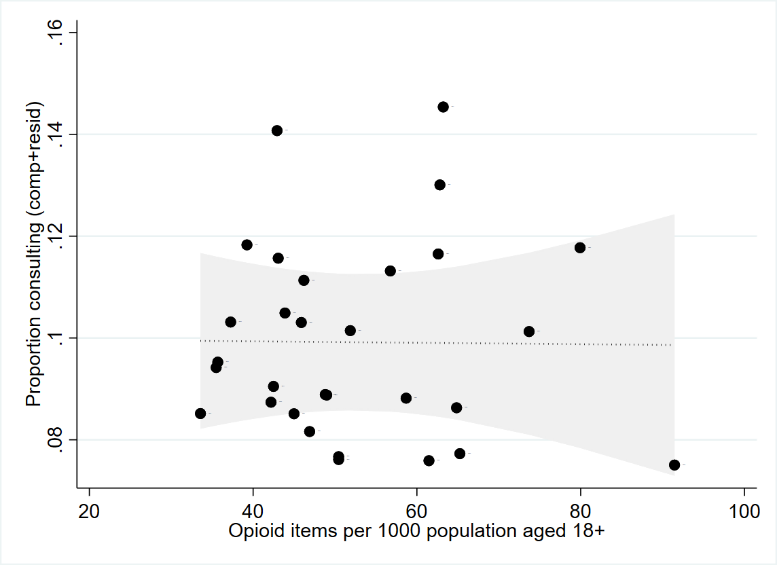** | **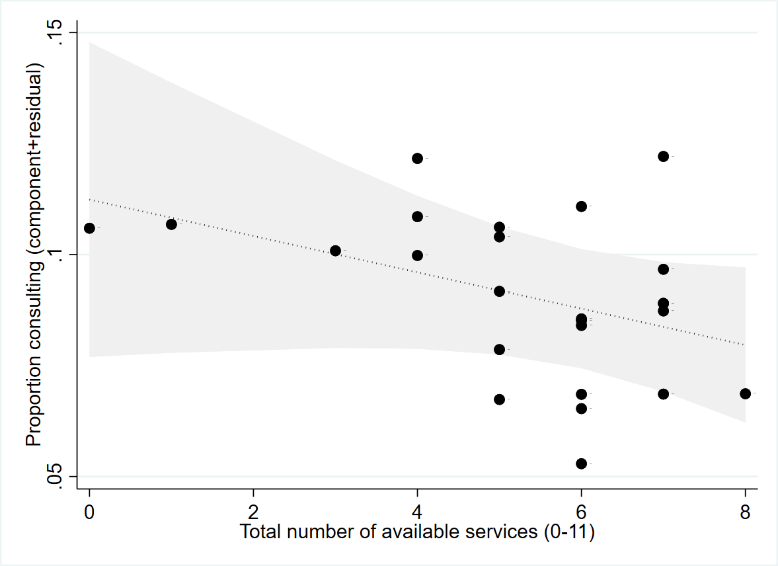** |
| LR χ^2^(1)=0.09, p=0.7616 | LR χ^2^(1)=2.66, p=0.1030 (n=25 practices) |

| **vs clinical decision support systems used** |
| --- |
| **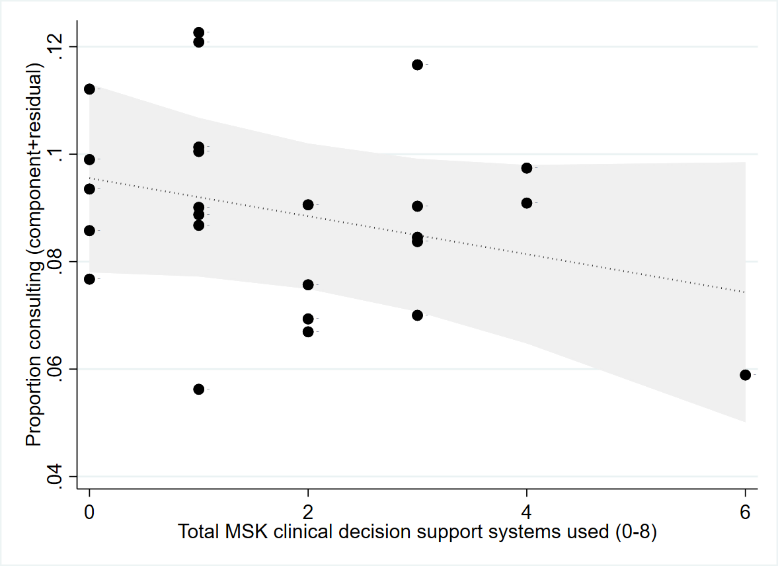** |
| LR χ^2^(1)=2.24, p=0.1348 |

**D5. Sensitivity analysis 2 – Replacement of single annual prevalence estimate with 3-year average annual prevalence estimate**

| **Single annual prevalence estimate** | **Average of annual prevalence estimates over 3 years** |
| --- | --- |
| **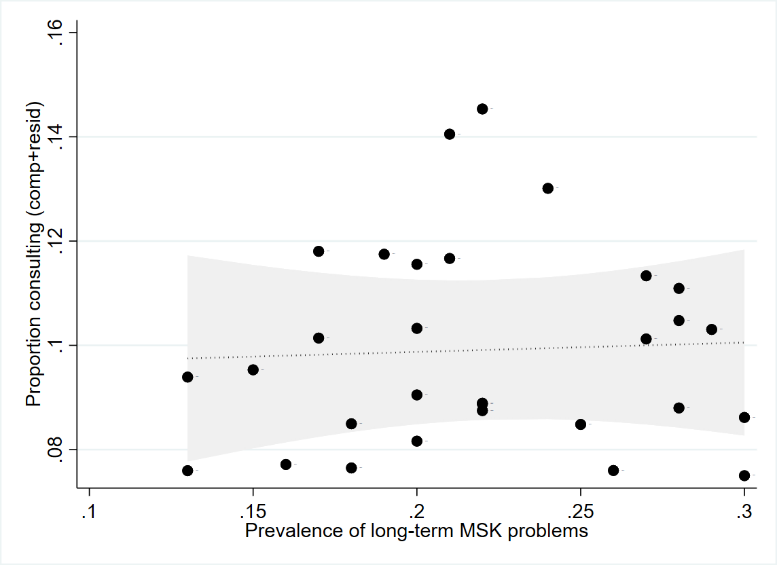** | **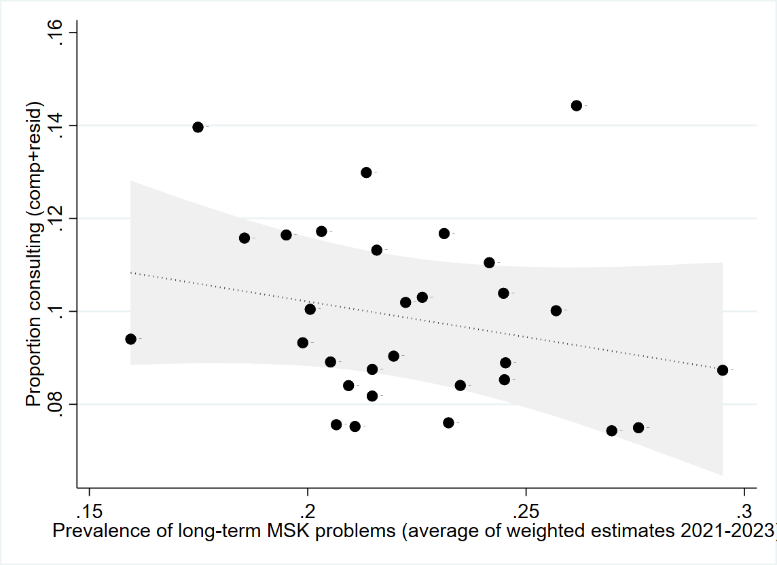** |
| LR χ^2^(1)=0.09, p=0.7616 | LR χ^2^(1)=2.66, p=0.1030 (n=25 practices) |

**D6. Sensitivity analysis 3 – Additional adjustment for recorded number of appointments per 1000 patients as a proxy for completeness of recording consultations**

| **Fig 1A** | **with additional adjustment** |
| --- | --- |
| **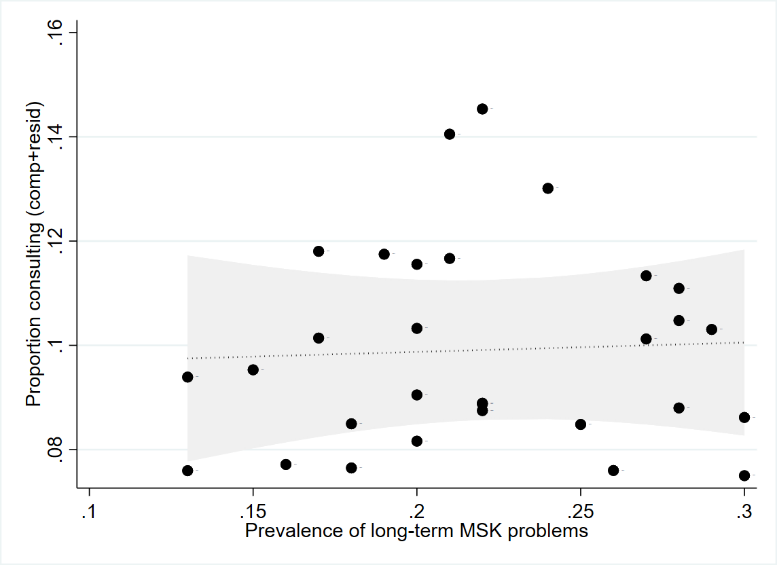** | **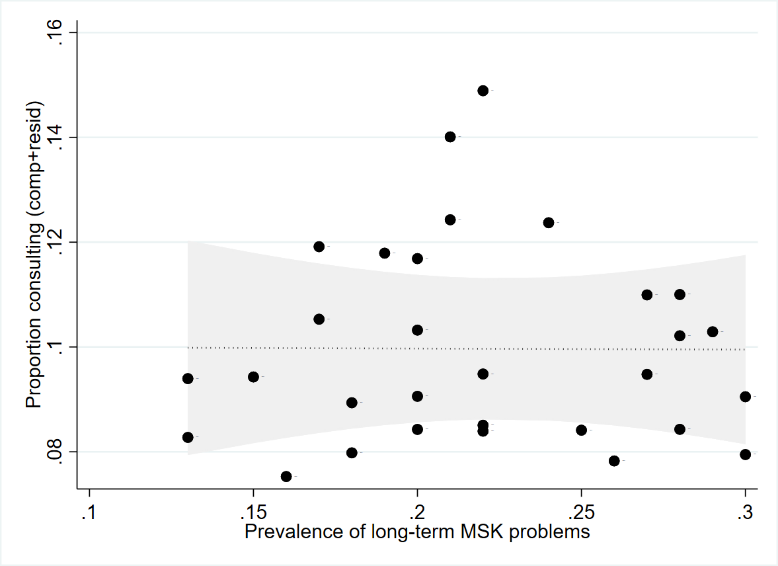** |

| **Fig 1B** | **with additional adjustment** |
| --- | --- |
| **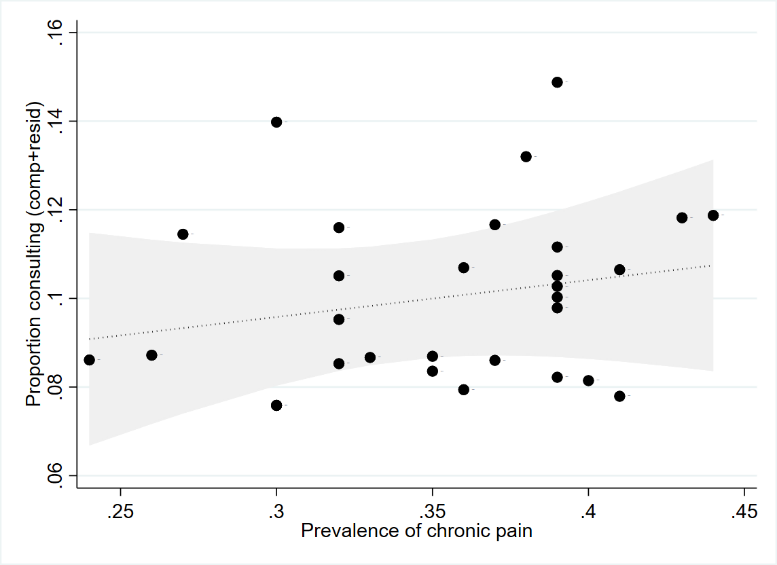** | **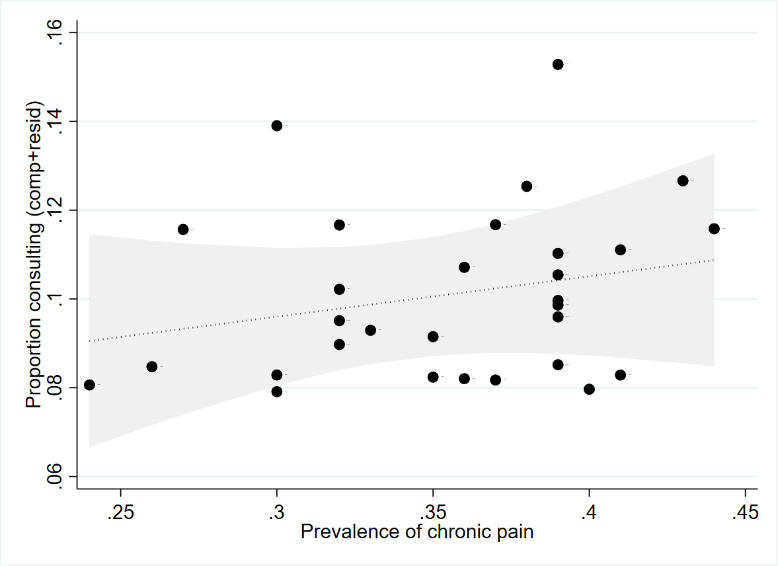** |

| **Fig 1C** | **with additional adjustment** |
| --- | --- |
| **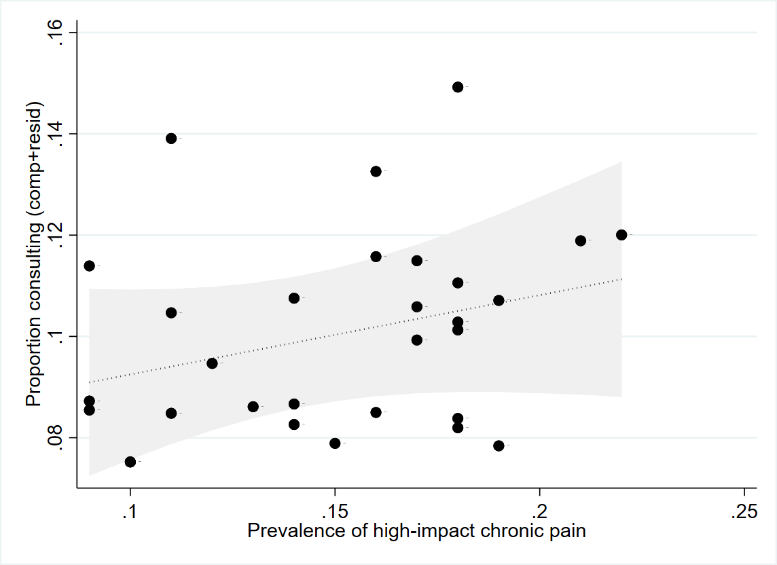** | **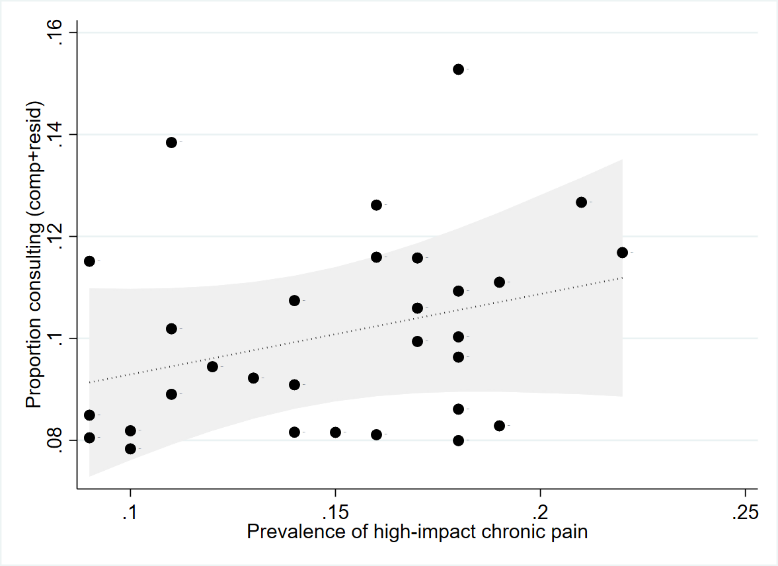** |

**D7. Summary table of associations with covariates**

|  | **Observed range** | **Dependent variable: Proportion consulting (Total)** | **Dependent variable: Proportion consulting (after excluding IA)** |
| --- | --- | --- | --- |
| **Registered population characteristics** | | | |
| Female (%)§ | 43-53 | LR χ^2^(1)=5.87, p=0.0154 | LR χ^2^(1)=1.44, p=0.2305 |
| Aged 65+ years (%)§ | 11-34 | LR χ^2^(1)=0.35, p=0.5569 | LR χ^2^(1)=0.71, p=0.3978 |
| Aged 85+ years (%)§ | 1-5 | LR χ^2^(1)=0.07, p=0.7860 | LR χ^2^(1)=0.41, p=0.5229 |
| Black, Asian, Minority Ethnic background (%) | 1-29 | LR χ^2^(1)=1.75, p=0.1863 | LR χ^2^(1)=0.01, p=0.9190 |
| Practice weighted deprivation decile (1-10)† | 1-9 | LR χ^2^(1)=0.24, p=0.6261 | LR χ^2^(1)=2.43, p=0.1188 |
| **Practice organisation and performance characteristics** | | | |
| Total population size (n) | 3065-14783 | LR χ^2^(1)=0.79, p=0.3750 | LR χ^2^(1)=9.05, p=0.0026 |
| Total clinical staff FTE per 10,000 | 7-11 | LR χ^2^(1)=0.94, p=0.3316 | LR χ^2^(1)=0.06, p=0.8119 |
| QOF Overall achievement (max=635) | 542-635 | LR χ^2^(1)=0.62, p=0.4295 | LR χ^2^(1)=1.13, p=0.2877 |
| Positive experience (%) | 44-92 | LR χ^2^(1)=1.94, p=0.1641 | LR χ^2^(1)=0.58, p=0.4480 |
| CQC Overall Rating‡ | RI-O | **-** | **-** |
| Available services (0-11)†† | 0-8 | LR χ^2^(1)=4.91, p=0.0267 | LR χ^2^(1)=0.78, p=0.3778 |
| Clinical decision support systems used (0-8)†† | 0-6 | LR χ^2^(1)=2.68, p=0.1018 | LR χ^2^(1)=1.54, p=0.2153 |
| **Need/burden estimates** | | | |
| Prevalence of self-reported long-term MSK problem (%) | 13-30 | LR χ^2^(1)=0.06, p=0.7998 | LR χ^2^(1)=0.14, p=0.7052 |
| Prevalence of chronic pain (%) | 24-44 | LR χ^2^(1)=0.82, p=0.3642 | LR χ^2^(1)=1.66, p=0.1977 |
| Prevalence of high-impact chronic pain (%) | 9-22 | LR χ^2^(1)=1.85, p=0.1741 | LR χ^2^(1)=2.79, p=0.0949 |
| QOF Obesity prevalence (%) | 8-20 | LR χ^2^(1)=3.45, p=0.0633 | LR χ^2^(1)=0.03, p=0.8572 |
| Opioid items per 1000 patients | 34-91 | LR χ^2^(1)=0.00, p=0.9564 | LR χ^2^(1)=0.09, p=0.7616 |
| **CQC** Care Quality Commission; **FTE** Full Time Equivalent; **IA** Inflammatory arthritis; **LR** Likelihood ratio; **QOF** Quality and Outcomes Framework; **RI** Requires Improvement; **O** Outstanding  All LR values are compared with model containing length of recruitment period, recruiting in December, proportion of registered population female, proportion of registered population aged 65+ years  † Based on Index of Multiple Deprivation where 1=Most deprived, 10=Least deprived  ‡ 26 (87%) practices were rated as ‘good’, providing insufficient variation for further meaningful analysis  § Denominator – registered population aged 18+ years  †† Based on 25 practices responding to practice questionnaire | | | |
